# Integrated study of Quercetin as a potent SARS-CoV-2 RdRp inhibitor: Binding interactions, MD simulations, and *In vitro* assays

**DOI:** 10.1371/journal.pone.0312866

**Published:** 2024-12-03

**Authors:** Ahmed M. Metwaly, Esmail M. El-Fakharany, Aisha A. Alsfouk, Ibrahim M. Ibrahim, Eslam B. Elkaeed, Ibrahim. H. Eissa

**Affiliations:** 1 Pharmacognosy and Medicinal Plants Department, Faculty of Pharmacy (Boys), Al-Azhar University, Cairo, Egypt; 2 Protein Research Department, Genetic Engineering and Biotechnology Research Institute (GEBRI), City of Scientific Research and Technological Applications (SRTA-City), New Borg El-Arab City, Alexandria, Egypt; 3 Pharmaceutical and Fermentation Industries Development Centre (PFIDC), City of Scientific Research and Technological Applications (SRTA-City), New Borg Al-Arab, Alexandria, Egypt; 4 Pharos University in Alexandria, Alexandria, Egypt; 5 Department of Pharmaceutical Sciences, College of Pharmacy, Princess Nourah Bint Abdulrahman University, Riyadh, Saudi Arabia; 6 Biophysics Department, Faculty of Science, Cairo University, Giza, Egypt; 7 Department of Pharmaceutical Sciences, College of Pharmacy, AlMaarefa University, Riyadh, Saudi Arabia; 8 Pharmaceutical Medicinal Chemistry & Drug Design Department, Faculty of Pharmacy (Boys), Al-Azhar University, Cairo, Egypt; Saveetha University - Poonamallee Campus: SIMATS Deemed University, INDIA

## Abstract

To find an effective inhibitor for SARS-CoV-2, Quercetin’s chemical structure was compared to nine ligands associated with nine key SARS-CoV-2 proteins. It was found that Quercetin closely resembles Remdesivir, the co-crystallized ligand of RNA-dependent RNA polymerase (RdRp). This similarity was confirmed through flexible alignment experiments and molecular docking studies, which showed that both Quercetin and Remdesivir bind similarly to the active site of RdRp. Molecular dynamics (MD) simulations over a 200 ns trajectory, analyzing various factors like RMSD, RG, RMSF, SASA, and hydrogen bonding were conducted. These simulations gave detailed insights into the binding interactions of Quercetin with RdRp compared to Remdesivir. Further analyses, including MM-GBSA, Protein-Ligand Interaction Fingerprints (ProLIF) and Profile PLIP studies, confirmed the stability of Quercetin’s binding. Principal component analysis of trajectories (PCAT) provided insights into the coordinated movements within the systems studied. *In vitro* assays showed that Quercetin is highly effective in inhibiting RdRp, with an IC_50_ of 122.1 ±5.46 nM, which is better than Remdesivir’s IC_50_ of 21.62 ±2.81 μM. Moreover, Quercetin showed greater efficacy against SARS-CoV-2 *In vitro*, with an IC_50_ of 1.149 μg/ml compared to Remdesivir’s 9.54 μg/ml. The selectivity index (SI) values highlighted Quercetin’s safety margin (SI: 791) over Remdesivir (SI: 6). In conclusion, our comprehensive study suggests that Quercetin is a promising candidate for further research as an inhibitor of SARS-CoV-2 RdRp, providing valuable insights for developing an effective anti-COVID-19 treatment.

## 1. Introduction

As of March 3^rd^, 2024, the World Health Organization (WHO) officially confirming an overwhelming total of 774,834,251 confirmed infections and a distressing toll of 7,037,007 recorded deaths attributed to the SARS-CoV-2 [1]. Despite the administration of 13.59 billion vaccine doses worldwide, the resilient nature of the virus persists, continuing to afflict populations on a widespread scale [1]. These disconcerting statistics stand as a reminder of the challenge faced by scientists across the globe. The relentless spread of the virus underscores the need for an urgent and collaborative effort from the global scientific community to uncover effective treatments.

The last two decades have witnessed a substantial increase in the adoption of Computer-Aided Drug Discovery (CADD) methods within the realm of pharmaceutical research [2, 3]. The widespread integration of CADD can be credited to several pivotal factors. Firstly, the ability to discern the precise 3D structures of numerous protein targets [4]. Secondly, the notable advancements in the capabilities of both computer software and hardware [5, 6]. Lastly, an enhanced understanding of the principles governing Structure-Activity Relationship and Quantitative Structure-Activity Relationship [7, 8]. Consequently, CADD techniques have been deployed to investigate diverse pharmacodynamic properties, facilitating the identification of relationships between chemical structure and activity. The CADD methodologies encompass pharmacophore identification [9], homology modeling [10], drug-complex interaction [11, 12], molecular docking [13], MD simulations [14], and structure similarity [15].

Throughout the history, nature has been indispensable in meeting fundamental human needs. Beyond offering remedies for medical treatments, it has been a source of sustenance and a key contributor to the formulation of various essential products [16, 17]. Flavonoids are naturally occurring phytochemicals recognized for their antiviral properties at multiple stages of viral infection, including viral entry, replication, and protein translation [18, 19]. These activities extending against a wide range of viral infections including SARS [20], hepatitis [21], influenza [22], and herpes [23]. Quercetin is a prominent member of flavonoids that has demonstrated strong antiviral activity *in vitro* through multiple mechanisms of action [24]. Notably, quercetin appears to inhibit viral entry by interacting with membrane glycoproteins, such as NA of H1N1 [25]. Additionally, quercetin targets specific viral proteases critical for replication, including NS2, and NS5A of the HCV [26], M^pro^ of SARS [27].

In our pursuit of countering the COVID-19 threat, our teamwork implemented a multi-stage in-silico CADD strategy to discern natural inhibitors and recommend the most fitting candidate against specific SARS-CoV-2 enzymes. Within more than 300 antiviral natural metabolites, we identified potential inhibitors for the SARS-CoV-2 nsp10 [28], helicase [29], the SARS-CoV-2 M^pro^ [30, 31], and the SARS-CoV-2 PLpro [32]. Moreover, from an array of 3009 FDA-approved drugs, we highlighted the most promising inhibitors against the SARS-CoV-2 RdRp [33], SARS-CoV-2 PLpro [34], SARS-CoV-2 M^pro^ [35] and the SARS-CoV-2 nsp16-nsp10 2′-o-Methyltransferase Complex [36]. Furthermore, the most potential inhibitors of SARS-CoV-2 RdRp [37], Helicase [38], and PLpro [39] among 4924 African natural products were pointed. Additionally, we conducted an investigation of 5956 compounds sourced from traditional Chinese medicine, with the objective of uncovering potential natural inhibitors for the SARS-CoV-2 Helicase enzyme [40]. The Coronaviridae family consists of positive-sense, single-stranded RNA viruses that rely on the RdRp for replication and transcription. Since the RdRp is crucial for the infection process, it represents a promising target for the development of antiviral therapies [41, 42].

In this study, we thoroughly examined how effective Quercetin is against the RdRp of SARS-CoV-2. We used a mix of computer simulations and lab experiments to compare it with Remdesivir, a known RdRp inhibitor. Our computational simulations involved analyzing the structure of Quercetin, utilizing molecular similarity, flexible alignment studies in addition to how it interacts with the RdRp using various techniques (molecular docking, MD simulations for 200 ns, MM-GBSA, PLIP, ProLIF, and PCAT). Meanwhile, experimentally, we tested Quercetin’s ability to block SARS-CoV-2 RNA production using a commercial kit. We also directly tested its ability to combat the SARS-CoV-2 in cell cultures. This dual-pronged approach gives a solid understanding of how Quercetin might work against SARS-CoV-2, and how it compares to Remdesivir.

## 2. Results and discussions

### 2.1. Computational studies

#### 2.1.1. Molecular similarity of Quercetin against nine co-crystallized ligands of SARSCoV-2 proteins

**Fig 1:**
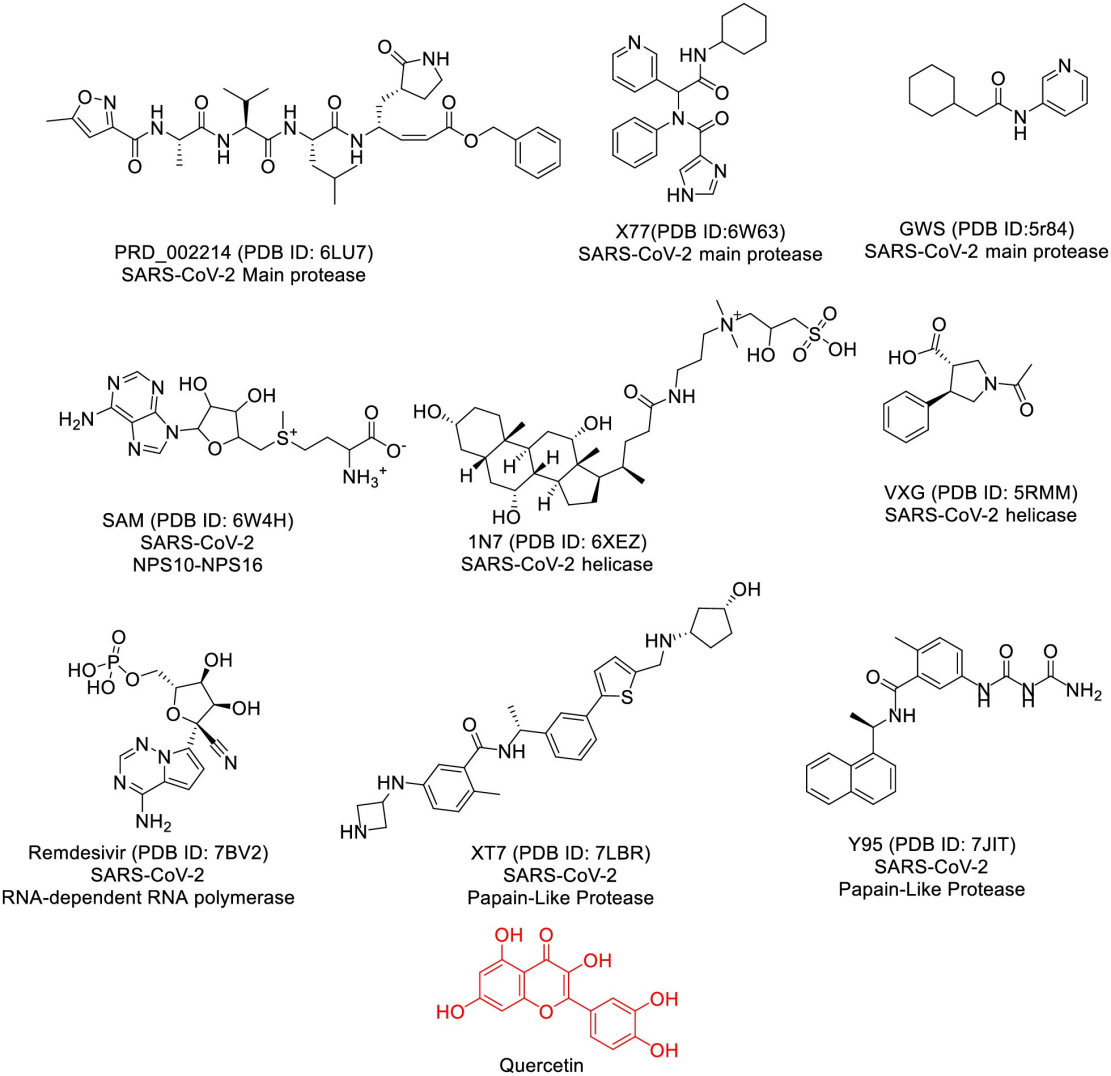
The co-crystallized ligands of SARS-Cov-2 proteins and Quercetin.

**Fig 2:**
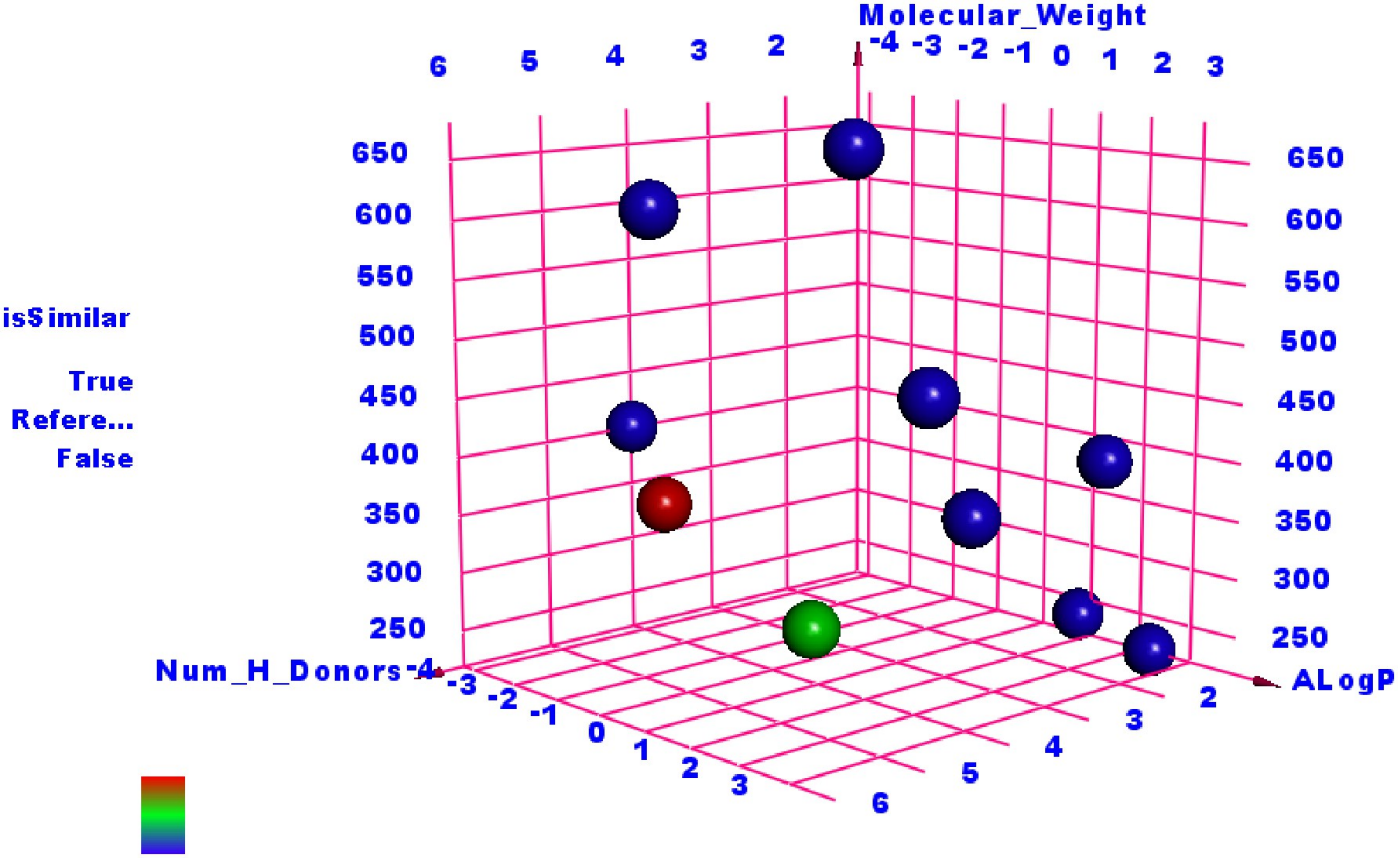
The analysis of similarity between the co-crystallized ligands of SARS-CoV-2 proteins and Quercetin (green ball) revealed notable parallels with Remdesivir (red ball).

Our focus in this investigation revolves around the co-crystallized ligand, a molecule that binding efficiently to a specific protein and inducing the crystallization of this protein, as a lead compound [43]. According to the principles of structure-activity relationship, compounds sharing similarities in chemical structures tend to exhibit akin biological activities through binding to the same receptor [44]. Our objective is to uncover potential structural resemblances that might impact binding affinity. Consequently, we conducted a molecular similarity study using Discovery studio software to scrutinize the chemical structure of Quercetin against nine co-crystallized ligands linked to vital proteins of SARS-CoV-2 (Fig 2). The structural and physicochemical properties of ligands, such as ALog p, molecular weight, hydrogen bond acceptors and donors, rotatable bonds, rings, aromatic rings, molecular fractional polar surface area (MFPSA), and minimum interaction distance, play a crucial role in understanding their behavior and effectiveness as potential drug candidates. ALog p reflects the hydrophobicity of a compound, influencing its interaction with hydrophobic or polar regions of a protein. Molecular weight affects a molecule’s size and absorption, with smaller molecules typically exhibiting better bioavailability. The number of hydrogen bond acceptors and donors determines the capacity for forming stabilizing interactions with target proteins, while rotatable bonds indicate molecular flexibility, impacting binding specificity. Aromatic rings contribute to π-π stacking interactions, enhancing ligand-protein affinity. MFPSA offers insights into the polarity and solubility of a compound, which can affect its permeability and bioavailability. Finally, the minimum distance between a ligand and a protein helps assess the fit and strength of binding interactions. Together, these parameters provide valuable information for evaluating the potential efficacy of ligands in inhibiting viral targets, such as SARS-CoV-2 proteins [45]. The molecular similarity study involves a comprehensive examination of the overall structures of both Quercetin and examined co-crystallized ligands, incorporating descriptors like steric, topological, electronic, and/or physical characteristics (Table 1 and Fig 1).

**Table 1:** Structural properties of Quercetin with the co-crystallized ligands of SARSCoV-2 proteins.

Compound	ALog p	M. Wt	HB acceptors	HB donors	Rotatable bonds	Rings	Aromatic rings	MFPSA	Minimum Distance
Quercetin	1.63	302	7	5	1	3	2	0.47	0
Remdesivir	-1.50	371	11	5	4	3	2	0.61	0.8215
PRD_002214	2.45	680	8	5	18	3	2	0.27	1.6171
GWS	2.17	218	2	1	3	2	1	0.18	1.4150
X77	2.62	403	4	2	6	4	3	0.22	1.2070
VXG	0.71	233	3	1	2	2	1	0.24	1.2830
1N7	0.23	631	8	6	12	4	0	0.25	1.5484
SAM	-4.25	399	9	4	7	3	2	0.48	1.0731
Y95	3.08	390	3	4	4	3	3	0.28	0.8981
XT7	3.87	504	5	5	9	5	3	0.22	1.3982

As shown in Fig 2, the findings indicated a significant resemblance between Quercetin and Remdesivir.

#### 2.1.2. Flexible alignment

**Fig 3:**
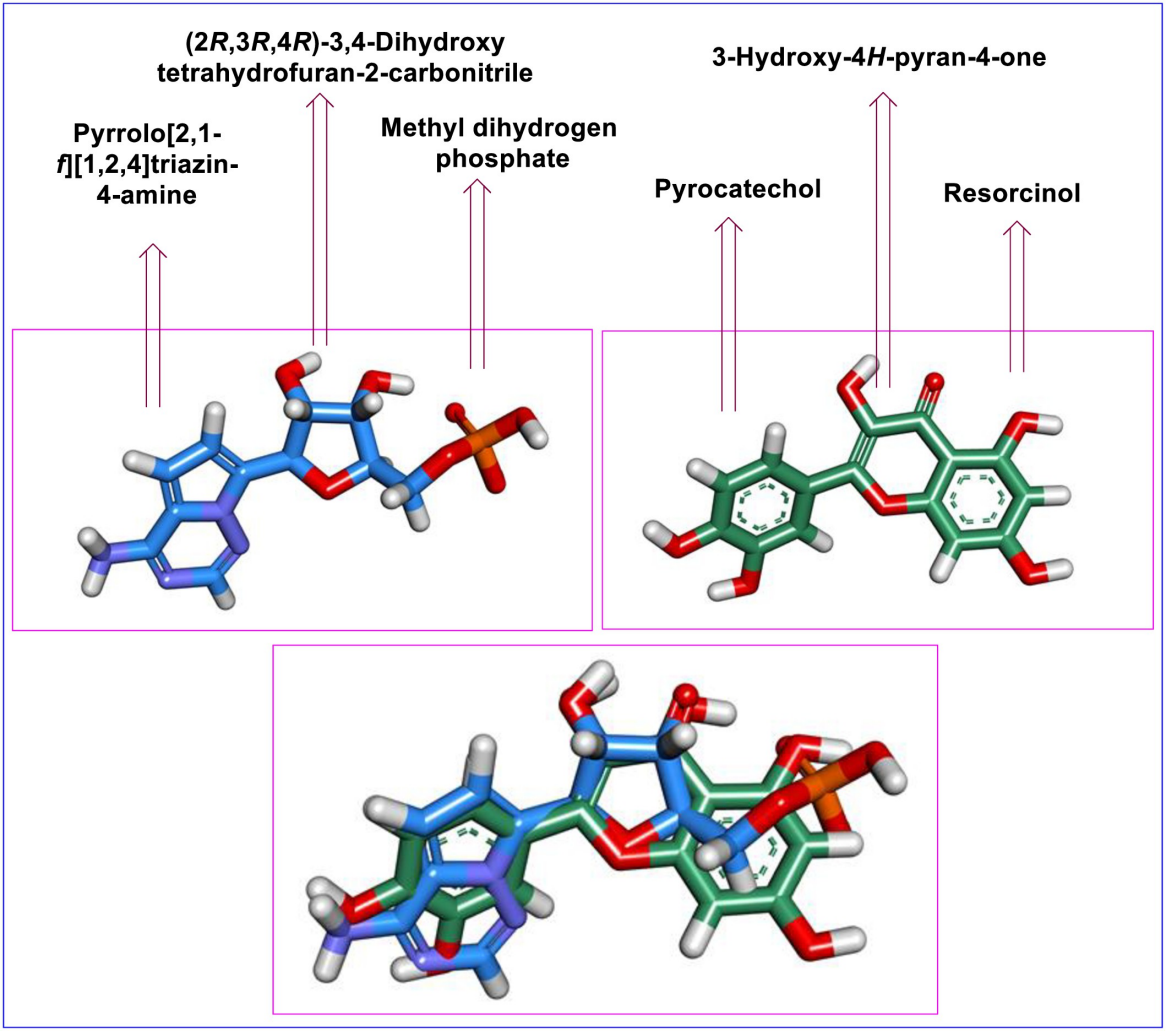
The 3D-flexible alignment of Quercetin (depicted in green) with Remdesivir (shown in blue).

To verify the indicated similarity, we performed a 3D-flexible alignment of Quercetin with Remdesivir following the flexible alignment procedure outlined in MOE2019. As depicted in Fig 3, it is evident that Quercetin exhibits a significant level of alignment with Remdesivir and both molecules have the same spatial orientation in space. In detail, the pyrocatechol moiety of Quercetin showed the same orientation as the 4-aminopyrrolo [2,1-*f*][1,2,4] triazine moiety of Remdesivir. Additionally, the 3-Hydroxy-4*H*-pyran-4-one moiety of Quercetin exhibited close orientation to the ((2*R*,3*S*,4*R*,5*R*)-5-cyano-3,4-dihydroxytetrahydrofuran-2-yl)methyl dihydrogen phosphate moiety of Remdesivir. Furthermore, the Resorcinol moiety of Quercetin showed the same orientation of Methyl dihydrogen phosphate moiety of Remdesivir.

#### 2.1.3. Docking studies

The examination of structural similarity revealed significant resemblances between Quercetin and Remdesivir, the designated ligand for the SARS-CoV-2 RNA-dependent RNA polymerase (RdRp). This similarity was validated through a flexible alignment study. Our subsequent step entails conducting molecular docking studies to explore whether Quercetin can effectively bind within the active site of RdRp, mimicking the binding mode observed with Remdesivir according to the previously published data [46, 47].

**Fig 4:**
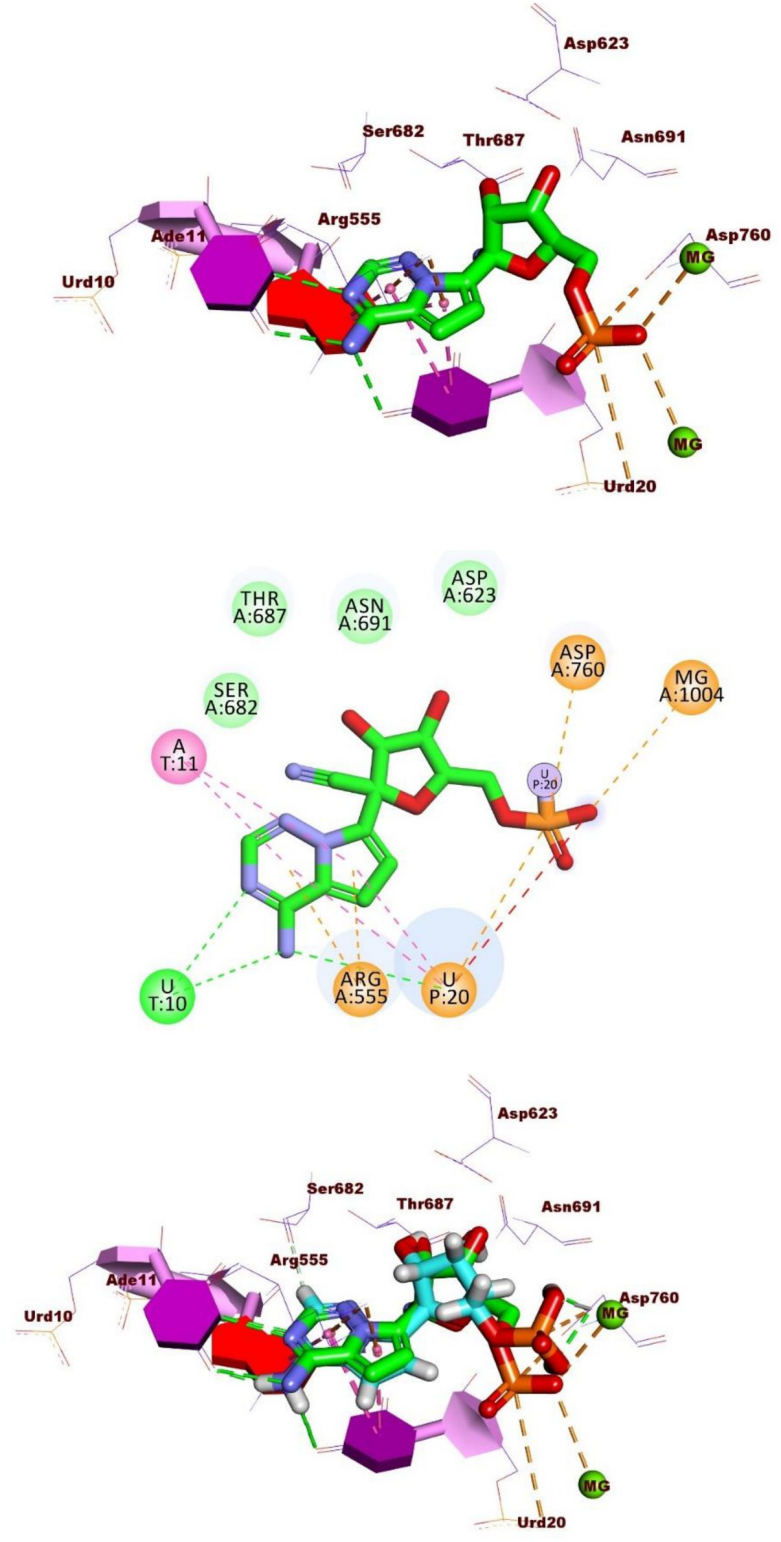
Interactions of Remdesivir in the active site of the RdRp A) 3D form and B) 2D form. C) The comparison of binding modes of docked (in green) and original (in cyan) co-crystallized ligand (Remdesivir).

Remdesivir showed a binding energy of -23.74 kcal/mol, involving three hydrogen bonds, four hydrophobic contacts, and seven electrostatic interactions. The 4-aminopyrrolo [2,1-*f*][1,2,4] triazine group occupied the RdRp’s active site, forming three hydrogen bonds with Urd10 and Urd20. In addition, it was engaged in four hydrophobic interactions with Ade11and Urd20. This moiety also created two electrostatic attractions with Arg555. Additionally, the phosphate group established five electrostatic attractions with Asp760, Urd20, and Mg1004 (Fig 4).

**Fig 5:**
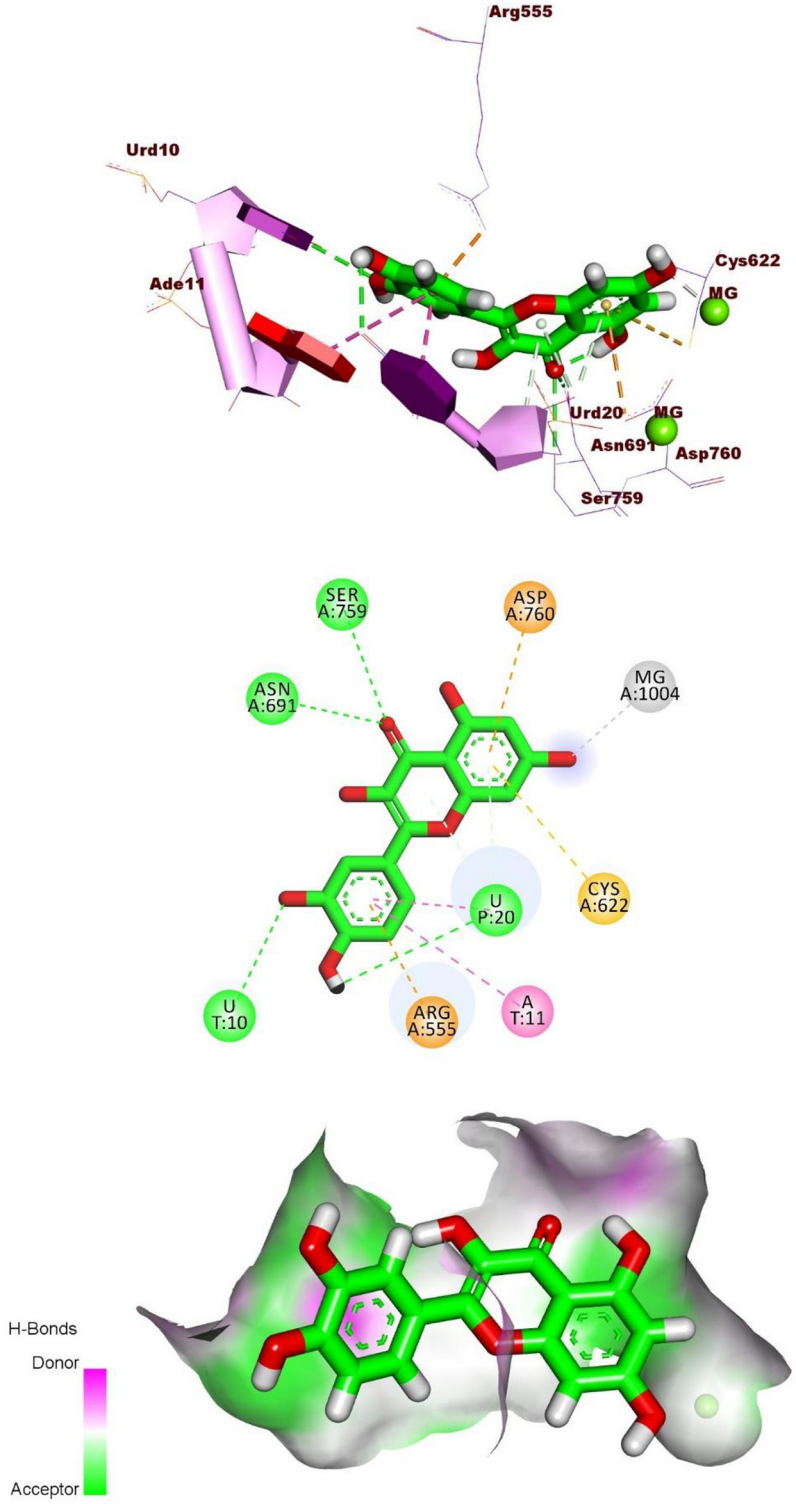
3D, 2D, and Surface mapping interactions of Quercetin in the active site of the RdRp.

Quercetin exhibited a binding mode similar to that of the reference molecule, Remdesivir, with a binding free energy of -20.29 kcal/mol. The pyrocatechol group was positioned in the RdRp active site, forming two hydrogen bonds with Urd10 and Urd20, along with two hydrophobic interactions with Ade11 and Urd20. Additionally, it formed an electrostatic interaction with Arg555. The 3,5,7-trihydroxy-4H-chromen-4-one group created two hydrogen bonds with Asn691 and Ser759. Also, it formed three electrostatic interactions with Asp760. Cys622, and Mg1004 (Fig 5).

**Fig 6:**
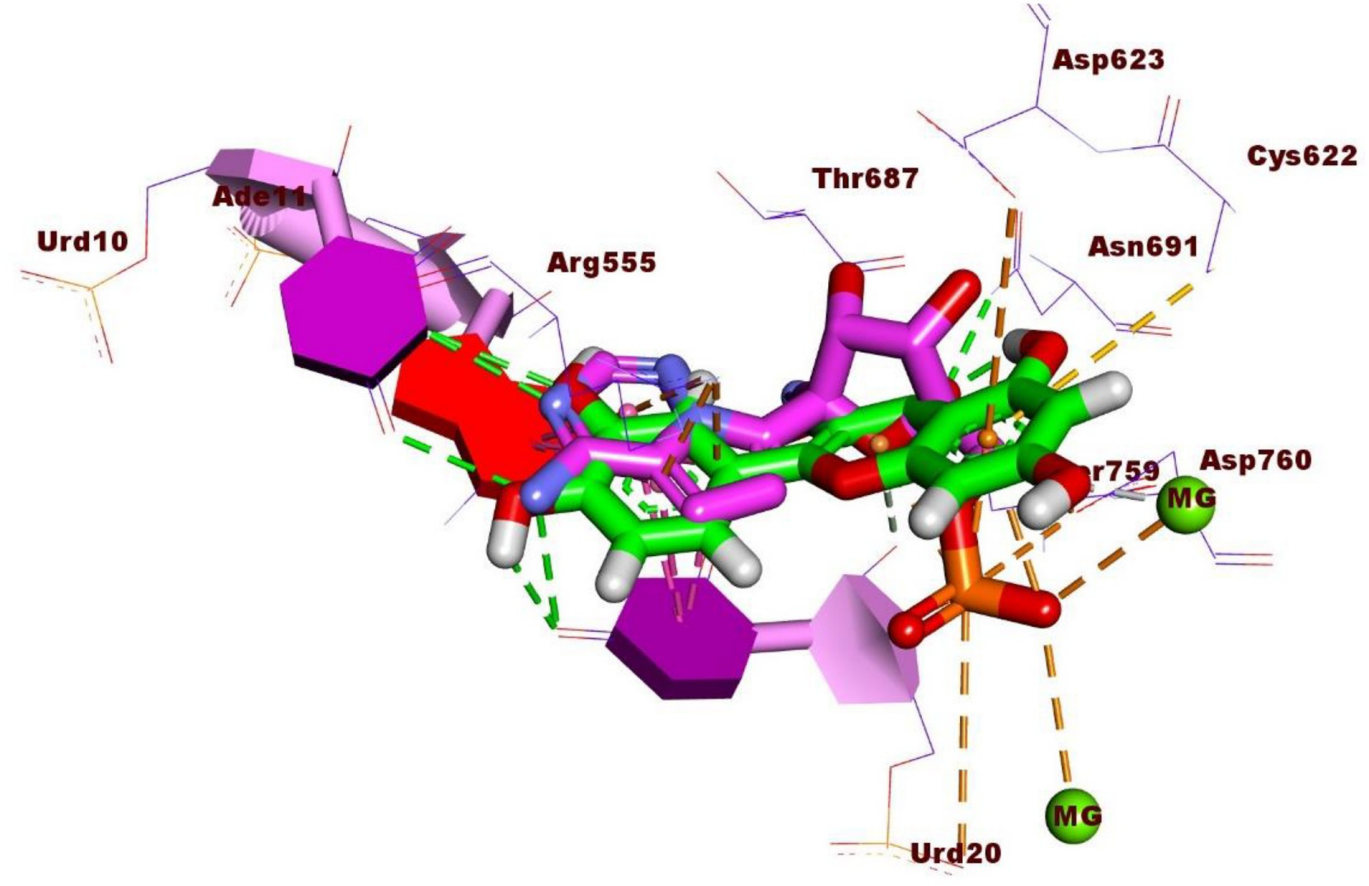
3D superimposition of Quercetin and Remdesivir in the active site of RNA-dependent RNA polymerase of SARS-CoV-2.

Fig 6 depicted the 3D superimposition of Quercetin and Remdesivir within the active site of the RdRp of SARS-CoV-2, offering a detailed comparison of their spatial arrangement. Notably, both molecules were observed to adopt a similar orientation within the active site, suggesting potential overlapping binding interactions with the enzyme. This alignment underscores the possibility of Quercetin mimicking the binding mode of Remdesivir, which is crucial for understanding its potential as an inhibitor of viral RNA replication.

#### 2.1.4. Molecular dynamics, MD, simulations analysis

**Fig 7:**
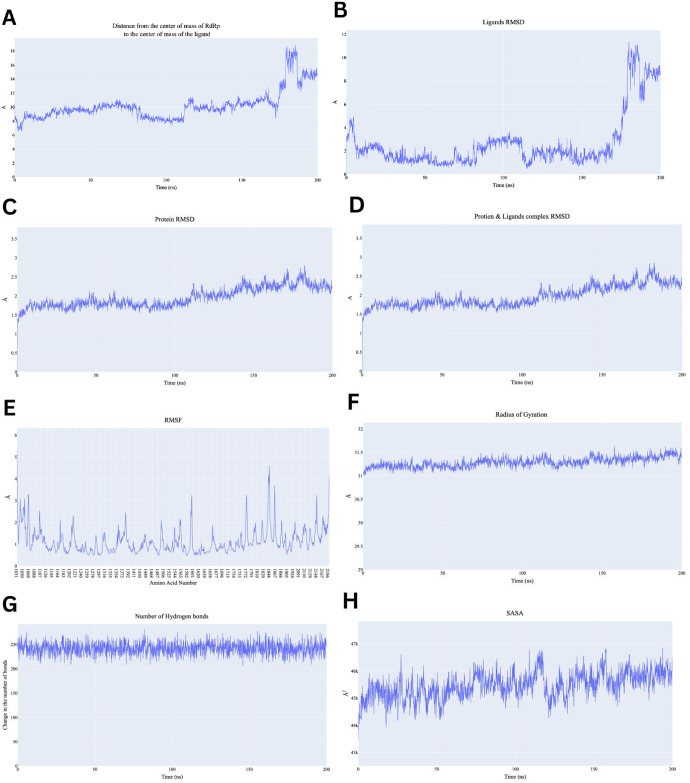
Analysis performed on the RdRp-Quercetin complex. A) Distance from the center of mass of Quercetin to RdRp, B) Quercetin RMSD, C) RdRp RMSD, D) the complex RMSD, E) protein RMSF based on the Carbon alpha atoms, F) Radius of Gyration for the protein, G) change in the number of H-bonds, H) the change in the SASA values.

Firstly, during the 200 nanoseconds (ns) of the production run showed Quercetin maintaining a steady distance around 8–10 Å during the initial 100 ns. However, after 100 ns, there is a gradual increase, and after 150 ns, the distance fluctuates sharply, reaching above 15 Å (Fig 7A). Similarly, the ligand RMSD started around 4 Å and remains stable for the first 100 ns, after which RMSD sharply increased reaching values above 10 Å (Fig 7B). These similar between COM and ligand RMSD suggest fluctuations in binding conformational of ligand in binding pocket. Furthermore, Protein backbone RMSD remained stable around 1.5–2.5 Å throughout the simulation, which is typical of a stable protein structure during MD simulations (Fig 7C). The stability of the protein RMSD indicates that the overall protein structure remains intact, even though the ligand exhibited significant fluctuations. Protein-Ligand Complex showed RMSD values starting around 1.5 Å and increases steadily until it stabilizes around 2–3 Å after 100 ns (Fig 7D). This increase might suggest that the complex undergoes conformational changes over simulation progression, but the system stabilizes after approximately 100 ns. Stabilization post 100ns indicates that although structural changes occurred, the complex remained stable over time. The RMSF shown in Fig 7E shows fluctuations across different amino acid residues, some regions shows fluctuations above 4 Å, especially around residues 1500–1800 and 2100–2180. Additionally, the Radius of gyration (Rg) fluctuates around 31 Å throughout the simulation reflecting protein maintaining its compactness and overall stable tertiary structure of conformation (Fig 7F). The SASA fluctuates between 43000 Å^2^ and 47000 Å^2^ suggesting minor changes in protein folding or ligand binding that may expose or hide different areas of the protein surface (Fig 7H). Finally, the number of hydrogen bonds remained stable fluctuating between 200 and 250 bonds during the simulation indicating that despite conformational changes and fluctuations in the system, the hydrogen bonding network remains intact and stable (Fig 7G). To sum up, the RdRp showed structural stability throughout the simulation, with stable RMSD and Rg values. However, the ligand appears to become unstable after the 100 ns mark, as indicated by the increased distance from the protein and elevated RMSD. The increase in SASA and fluctuations in the RMSF plot indicate that certain regions of the protein exhibited degree of flexibility, potentially corresponding to conformational changes in response to ligand.

**Fig 8:**
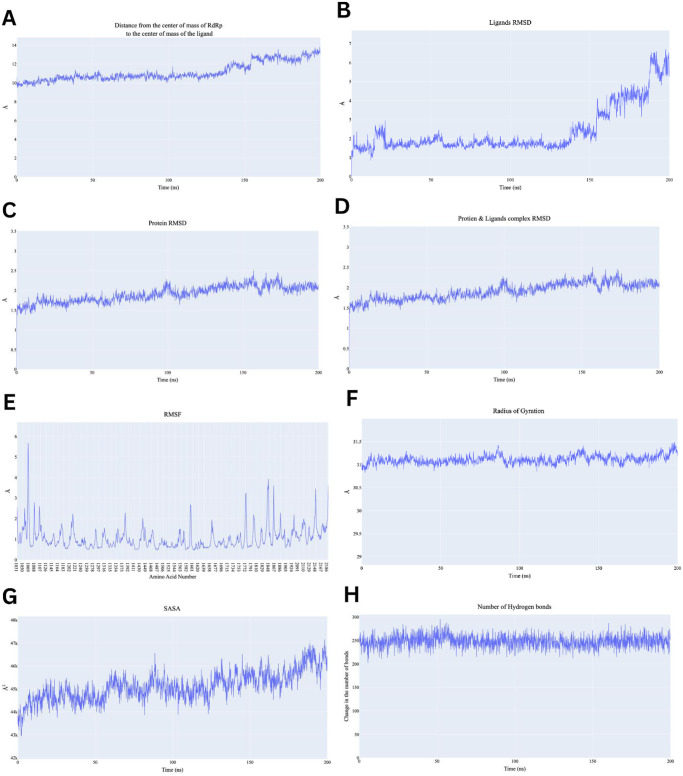
Analysis performed on the RdRp-Remdesivir complex. A) Distance from the center of mass of Remdesivir to RdRp, B) Remdesivir RMSD, C) RdRp RMSD, D) the complex RMSD, E) protein RMSF based on the Carbon alpha atoms, F) Radius of Gyration for the protein, G) the change in the SASA values, H) change in the number of H-bonds.

On the other hand, the distance between the center of mass between RdRp and Remdesivir fluctuated between 10 Å and 14 Å showing a gradual increase over time (Fig 8A). This indicates that while the ligand interacts with the protein initially, there is a slight movement or displacement over time. The increase after 100 ns suggests potential loosening or structural adjustments in the binding site. The Remdesivir RMSD increases significantly after around 100 ns, from around 1 Å to above 6 Å (Fig 8B). This suggests that the ligand is moving or exhibiting structural rearrangements within the binding site. The Root Mean Square Deviation (RMSD) of protein-ligand complex starts at 1.5 Å and gradually increases till it reaches 2.5 Å (Fig 8D). Showing that complex underwent conformational changes. Slight fluctuations after 150 ns could also indicate minor adjustments in the binding mode or conformational changes. Additionally, the protein backbone shows a stable RMSD that gradually increasing from 1.5 Å to 2.0 Å (Fig 8C). The steady RMSD indicates that the protein structure itself remains stable throughout the simulation with minimal deviations. RMSF gives insight into the flexibility of individual residues (Fig 8E). RMSF shows that most regions of RdRp remain relatively stable, with fluctuations typically below 2 Å. However, there are notable peaks around residue numbers 1000, 1600, and 2200, indicating regions of higher flexibility. Regarding protein compactness, radius of gyration and SASA are relatively stable fluctuating around 31 Å and 45000 Å^2^ indicating compactness of the protein does not change significantly during the simulation (Fig 8F and 8G). Finally, number of hydrogen bonds fluctuates between 200 and 280, which is a good indication of strong stability (Fig 8H). The consistent presence of hydrogen bonds indicates robust intermolecular interactions between Remdesivir and RdRp, which are key to maintaining the complex’s stability.

Comparing behaviour of two compounds, centre of mass between RdRp and Quercetin started at around 8 Å, remaining quite stable and fluctuating between 8 Å and 10 Å throughout the simulation. While centre of mass between Remdesivir fluctuates more ranging from 10 Å to 14 Å, showing a gradual increase over time. Therefore, Quercetin seems to bind more tightly and consistently to RdRp, while Remdesivir shows some movement away from its initial binding site. Quercetin’s stronger binding could suggest a more stable interaction with RdRp. Accordingly, Quercetin ligand RMSD remains fairly stable, below 2 Å throughout the simulation. On the other hand, Remdesivir’s RMSD shows significant increases after around 100 ns, rising to more than 6 Å, indicating Remdesivir exhibiting larger conformational changes. Quercetin appears to be more conformationally stable within its binding site compared to Remdesivir, which shows significant movement and possible dissociation.

Regarding protein backbone behaviour for both Quercetin and Remdesivir both ligands maintained overall structural integrity of RdRp, but the Remdesivir-bound RdRp system shows slightly more deviations. Collectively, protein-ligand complex the RMSD of the Quercetin-RdRp complex showed lower fluctuations, staying between 1.5 Å to 2.5 Å, with no significant increases or large deviations, indicating a stable complex. While Remdesivir-RdRp complex showed similar RMSD pattern, but with slightly larger deviations, particularly after 150 ns, indicating that the overall complex is less stable than the Quercetin complex. In conclusion, both complexes are relatively stable, but the Quercetin complex appears to be more structurally conserved compared to Remdesivir. Moreover, radius of gyration (Rog) of both ligands maintained overall compactness of RdRp, suggesting that neither ligand causes significant unfolding or structural changes in the protein. Same observation was carried down for SASA; as both ligands maintained consistent solvent exposure with Remdesivir showing slight fluctuation suggesting more structural rearrangements that expose more of the protein to the solvent.

#### 2.1.5. Molecular mechanics-generalized born surface area, MM-GBSA, analysis

**Fig 9:**
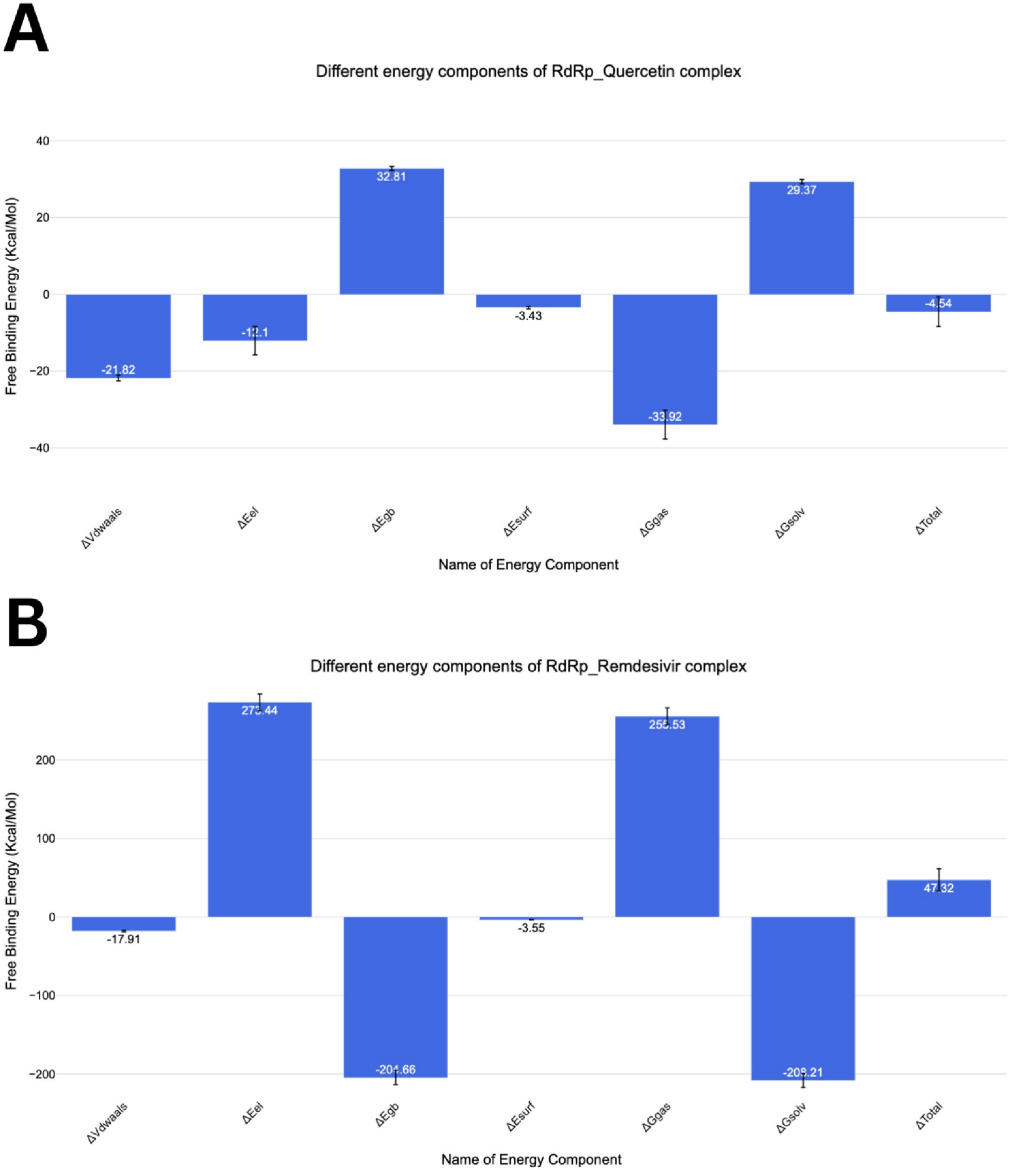
The energetic parameters obtained from MM-GBSA investigations, along with their respective numerical values, while the bars indicate the standard deviations associated with each measurement.

**Fig 10:**
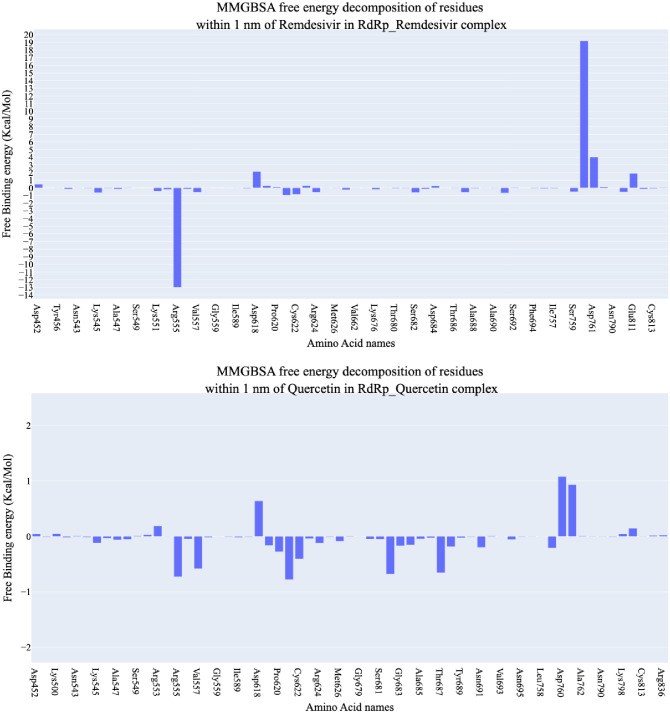
The breakdown of binding free energies for the complexes involving RdRp-Quercetin and RdRp-Remdesivir.

Fig 9 illustrates the computed binding free energy components utilizing the MM-GBSA technique. The calculated binding energies for RdRp with Quercetin and Remdesivir are **47.32** and **-4.54** and kcal/mol, respectively, indicating a slightly better interaction for Quercetin (Fig 9A & 9B). Notably, both compounds display significant Van der Waals contacts, with values of -17.91 kcal/mol for Remdesivir and– 21.82 kcal/mol for Quercetin. Moreover, Remdesivir exhibits superior electrostatic interactions 273.44 kcal/mol compared to Quercetin -12.10 kcal/mol. Fig 10A & 10B presents the decomposition analysis, specifically focusing on amino acids within a 4Å proximity or less to Quercetin or Remdesivir. For Remdesivir, these amino acids are Arg555 (-12.99 kcal/mol), Val557 (-0.55 kcal/mol), and Thr687 (-0.57 kcal/mol). On the other hand, Quercetin showed more interaction components. For instance, Arg555 (-0.72 kcal/mol), Val557 (-0.58 kcal/mol), Tyr619 (-0.16kcal/mol), Pro620 (-0.27 kcal/mol), Lys621 (-0.77 kcal/mol), Cys622 (-0.4 kcal/mol), Ser682 (-0.67kcal/mol), Thr687 (-0.65kcal/mol), and Ser759 (0.21kcal/mol).

#### 2.1.6. Protein-Ligand Interaction Fingerprints (ProLIF) analysis

**Fig 11:**
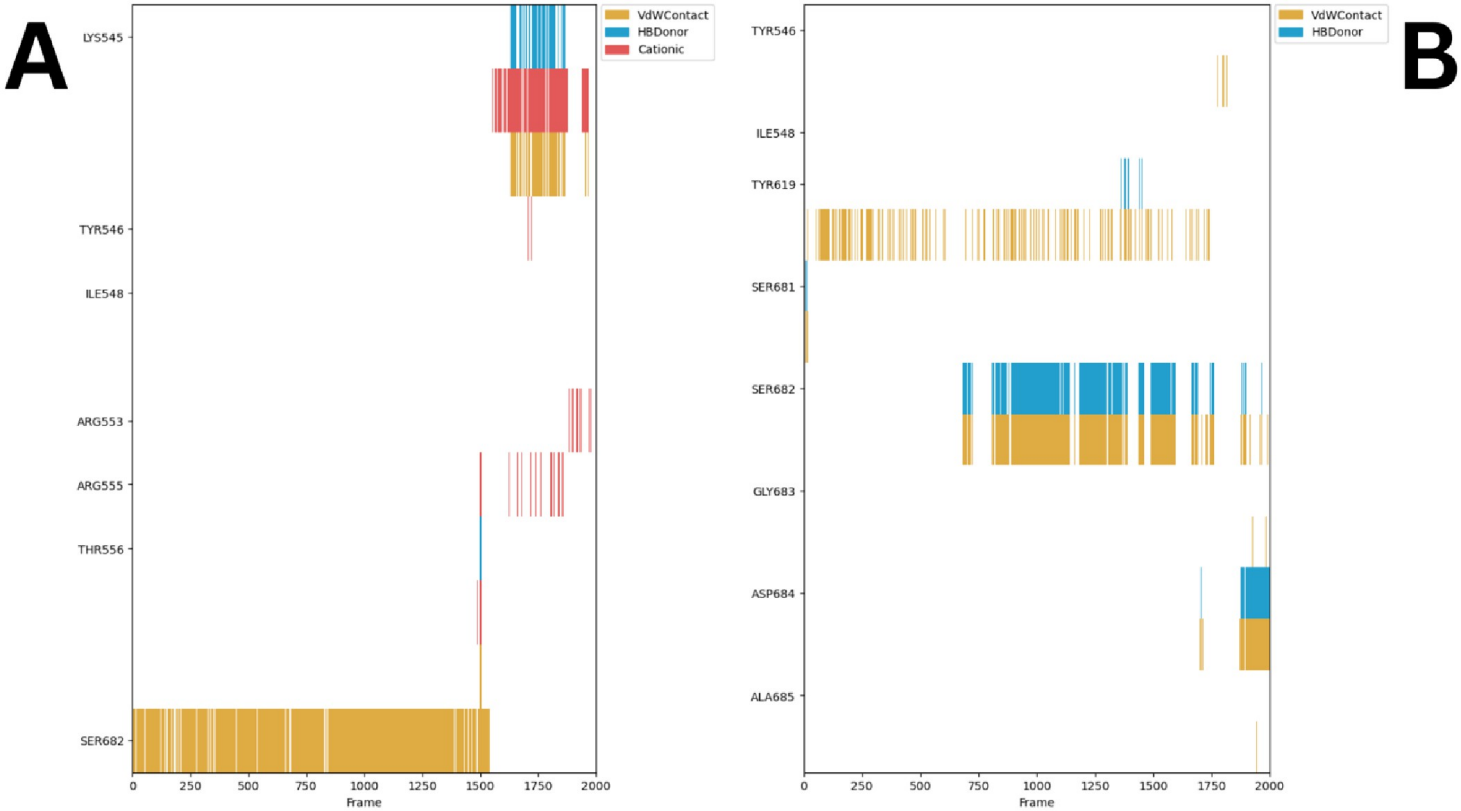
The amino acids grouped according to their interactions with A) Remdesivir (occurring in at least 50% of cases) and B) Quercetin (occurring in at least 75% of cases). Additionally, it presents their occurrence frequency throughout the entire simulation period, employing the ProLIF Python library.

A focused simulation analysis was conducted utilizing the ProLIF library studies to meticulously scrutinize the interacting amino acids in both the RdRp-Quercetin and RdRp-Remdesivir complexes. The objective of this investigation was to reveal the particular amino acids pivotal to the molecular interactions, thereby providing valuable insights into the binding interfaces of these complexes [48]. The results obtained from the ProLIF library demonstrate the binding interactions of Remdesivir (Fig 11A). The strong and consistent van der Waals interactions observed with SER682 for almost 75% of simulation run time, suggests that this residue plays a significant role in maintaining stable binding throughout the molecular dynamic’s simulation. Intermittent cation-π interactions at ARG555 and THR556 point to possible conformational changes or transient binding events. Importantly Lys545 showed capacity to bind to remdesivir through VdW contact, cation-π interactions, and HB donor interaction for the last 30% of simulation run time. In contrast, Quercetin exhibits a more dynamic interaction pattern, (Fig 11B) especially with **SER682** for approximately 62% of simulation run time, engaging through van der Waals and hydrogen bonds. The intermittent interactions observed with **TYR619** and **SER681** suggest a potential difference in binding stability between Quercetin and Remdesivir, affecting their respective inhibitory impacts on RdRp.

#### 2.1.7. Protein Ligand Interaction Profile (PLIP) analysis

**Fig 12:**
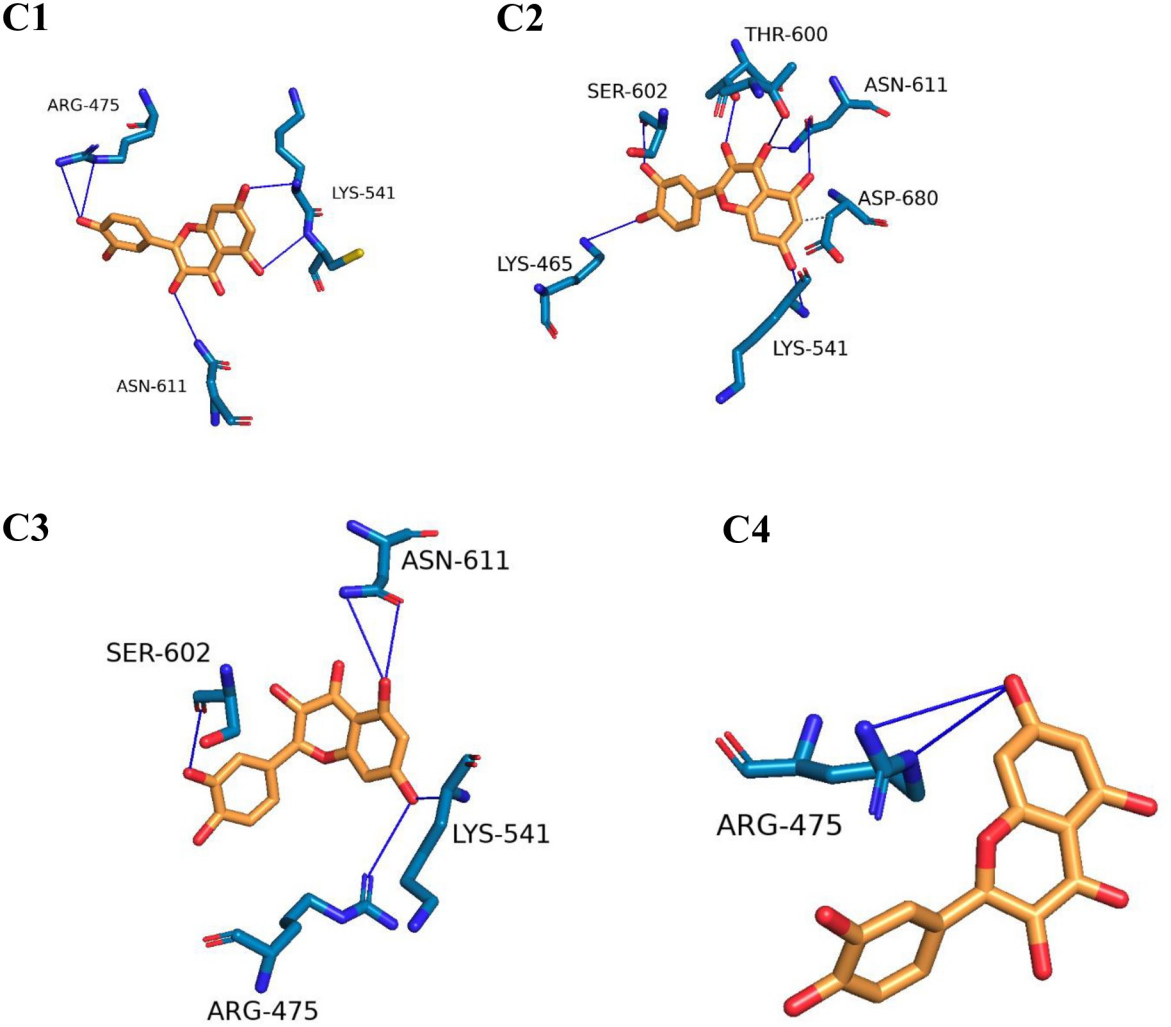
The interactions stemming from representative clusters identified by TTClust and their three-dimensional interactions with Quercetin. Grey dashed lines represent hydrophobic interactions, and blue solid lines depict hydrogen bonds. Quercetin is depicted using orange sticks, while the amino acids of the RdRp protein involved in these interactions are represented by blue sticks.

**Fig 13:**
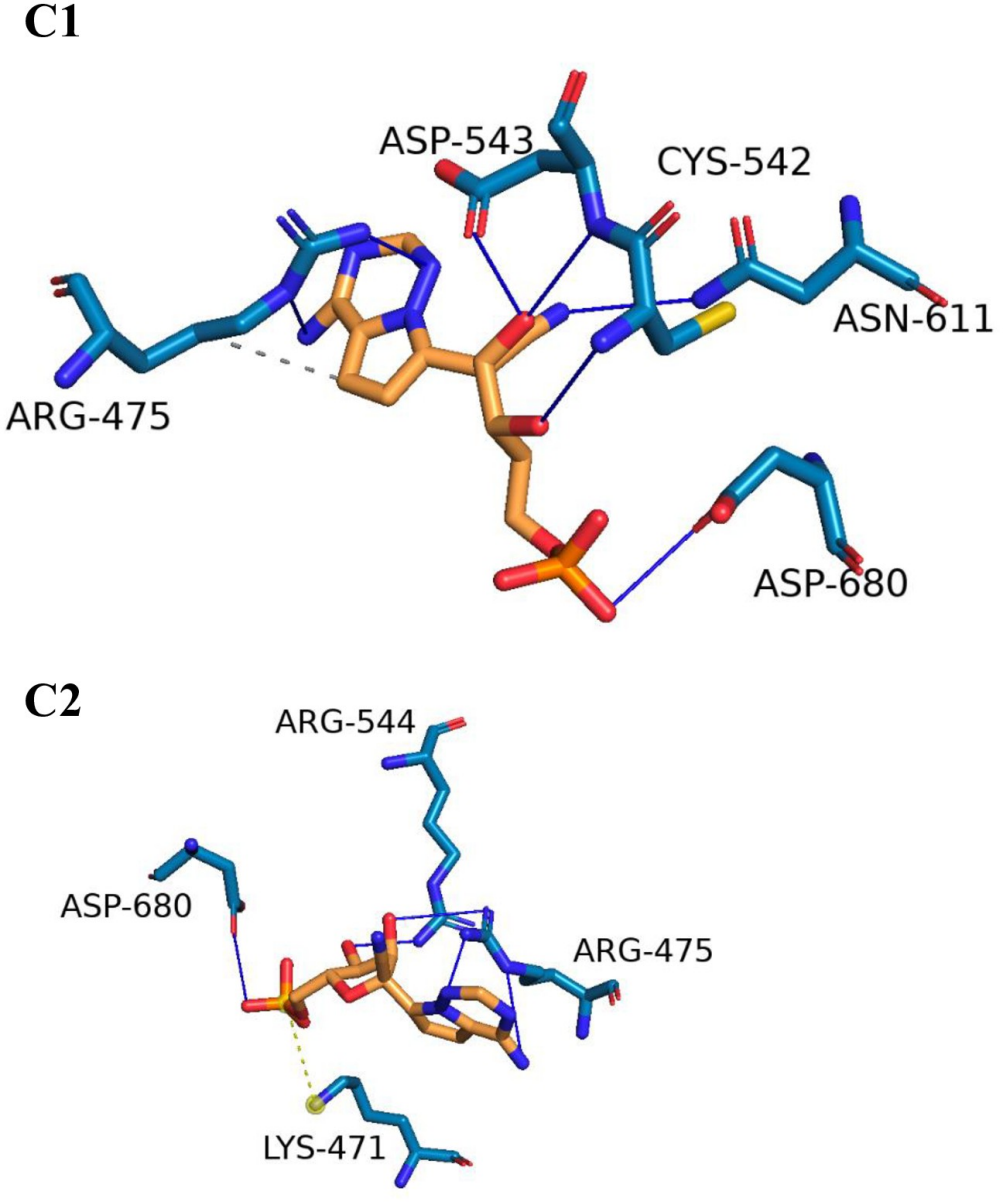
The interactions stemming from representative clusters identified by TTClust and their three-dimensional interaction with Remdesivir. Dashed grey lines represent hydrophobic interaction, dashed yellow lines denote salt bridges, and solid blue lines depict hydrogen bonds. Remdesivir is depicted using orange sticks, while the amino acids of the RdRp protein involved in these interactions are represented by blue sticks.

To visualize the three-dimensional binding interactions of the RdRp-Quercetin and RdRp-Remdesivir complexes, we utilized the PLIP tool to generate.pse files from representative frames [49]. Figs 12 and 13 present a visual representation of the interactions identified from clusters identified by TTClust, showcasing the distinct 3D interactions of Quercetin and Remdesivir with the RdRp. Notably, as depicted in Fig 12, Quercetin demonstrates engagement with four distinct clusters, displaying a multitude of interactions that similar to those observed with Remdesivir. The interaction analysis of Quercetin with RdRp reveals four main clusters of hydrogen bonding with key residues in chain A. Throughout the clusters, important interactions are observed with residues such as ARG475, LYS541, THR607, and ASN611. In Cluster 1, ARG475, LYS541, CYS542, and ASN611 are involved in significant interactions, highlighting their role in maintaining the binding stability of Quercetin within the active site. In Cluster 2, LYS465, THR600, and ASN611 emerge as important interacting residues. The repeated involvement of ASN611 and LYS541 indicates their consistent role in ligand stabilization. Cluster 3 continues to show strong involvement of ARG475 and LYS541, while ASN611’s interaction remains steady, indicating its central role in ligand recognition and stability. Finally, Cluster 4 underscores the importance of ARG475 and LYS541 in the Quercetin-RdRp interaction, suggesting a recurring pattern of hydrogen bonding that contributes to the ligand’s binding stability.

Conversely, as illustrated in Fig 13, Remdesivir exhibits interactions within two clusters, albeit with comparatively levels of engagement. This comparative analysis offers a comprehensive spatial understanding of the dynamic binding interactions of Quercetin and Remdesivir with the RdRp. ARG475 shows notable hydrophobic interactions with Remdesivir, specifically with a distance of around 3.95 Å. This interaction occurs consistently with the ligand’s atoms and suggests that ARG475 plays a key role in stabilizing the ligand within the binding site. ARG475 participates in hydrogen bonding with Remdesivir in both clusters, showing consistent interactions through both side chain and backbone atoms. This bond appears with distances ranging from 2.14 Å to 3.96 Å, highlighting its stabilizing role. CYS542 and ASP543 also form hydrogen bonds with Remdesivir, specifically in cluster 1. These bonds are relatively strong, with distances between 1.88 Å to 3.88 Å, contributing to the anchoring of Remdesivir in the binding site. ASN611 is involved in multiple hydrogen bonds, with distances around 2.91 Å, indicating its importance in ligand binding. Similarly, ASP680 in both clusters forms significant hydrogen bonds with Remdesivir, further highlighting its interaction importance. A salt bridge interaction is observed between LYS471 and Remdesivir, specifically with the phosphate group of Remdesivir. This salt bridge shows a distance of around 5.40 Å, demonstrating that LYS471 plays a crucial role in electrostatic interactions and ligand stabilization through its interaction with the phosphate moiety.

#### 2.1.8. Principal component analysis of trajectories (PCAT) analysis

**Fig 14:**
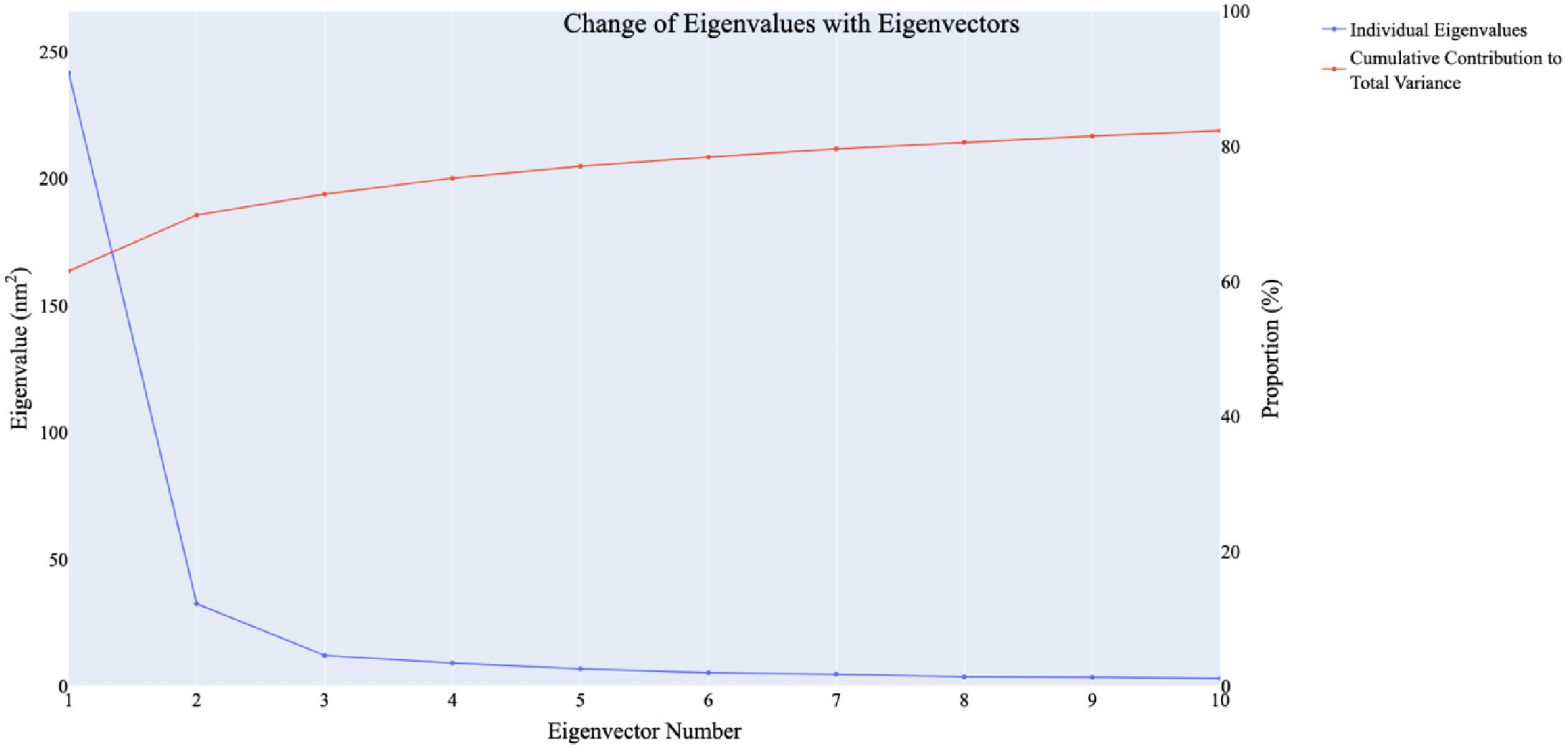
The change in eigenvalues with the increasing of the number of eigenvectors (blue), while the red line illustrates the cumulative variance retained by the eigenvectors.

Principal component analysis of trajectories (PCAT) was used to identify the coordinated movements within the system. Various metrics, detailed in the methodology section, were employed to determine the dimensionality of the reduced subspace. The scree plot revealed a significant inflection point at the fourth principal component (PC). As illustrated in Fig 14, the first eigenvector accounted for approximately 64% of the total variance, and together, the first four eigenvecto explained nearly 78% of the overall variation.

**Fig 15:**
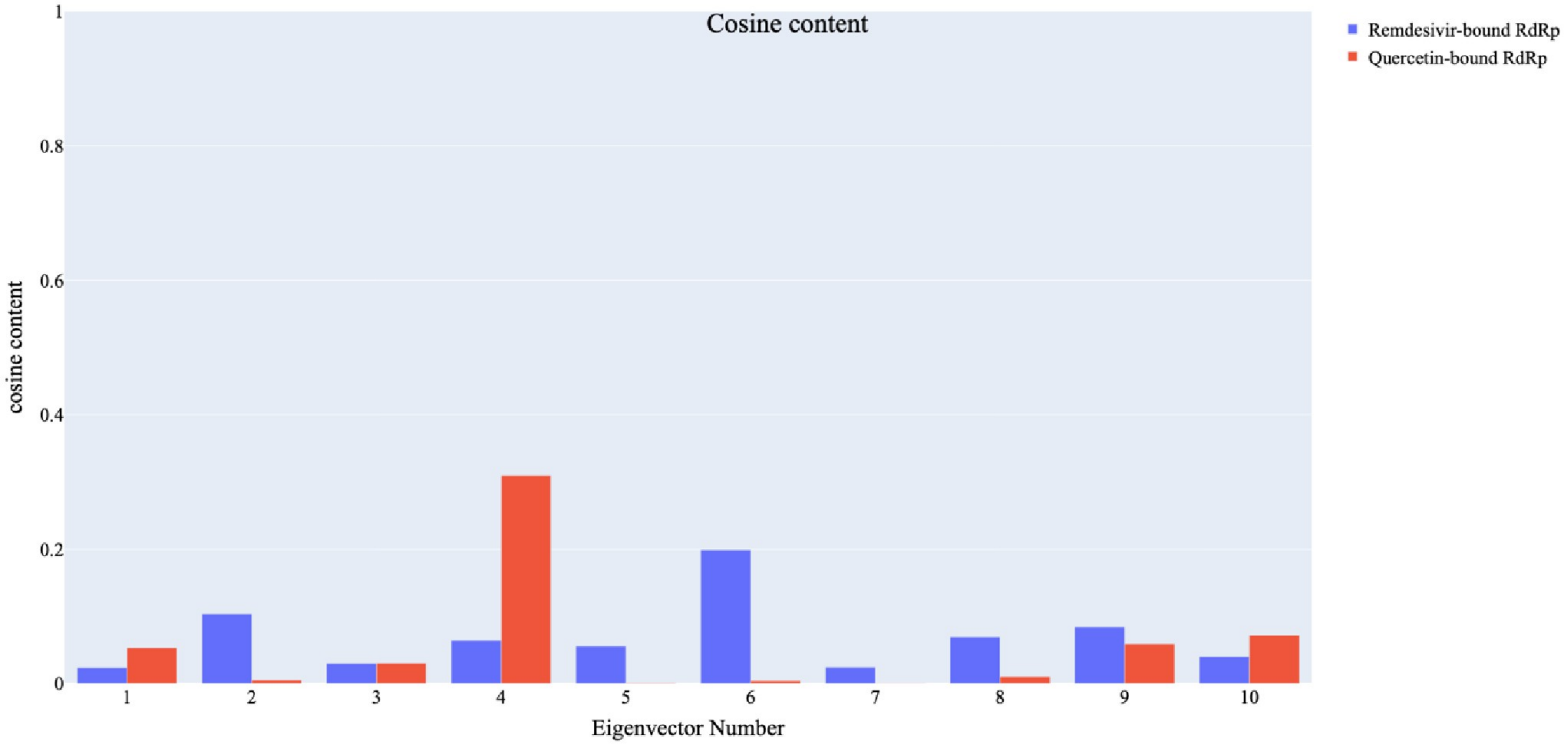
The cosine content values of the first ten eigenvectors for the two trajectories of the RdRp-Quercetin and RdRp-Remdesivir complexes.

To assess the level of randomness in the behaviour of the initial ten eigenvectors. The cosine content analysis (Fig 15) was used to assess the randomness of the eigenvectors. For the RdRp-Quercetin system, the fourth eigenvector had the highest cosine content (0.78), while the RdRp-Remdesivir system had notable contributions from the sixth eigenvector (cosine content 0.61). This analysis indicates that the two systems exhibit distinct dynamical behaviours. Eigenvectors with low cosine content signify concerted motions rather than random fluctuations.

**Fig 16:**
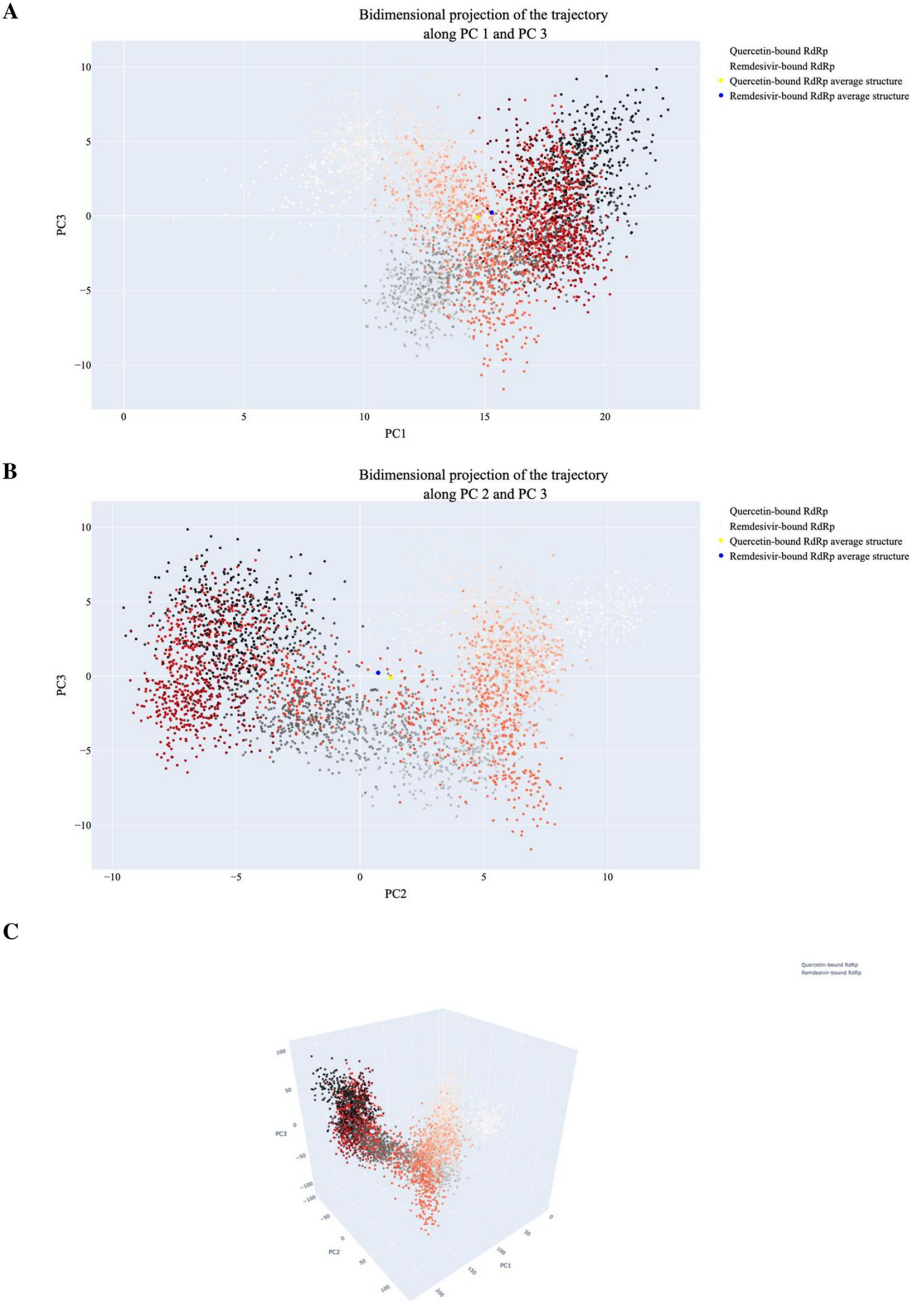
The projection of the RdRp-Quercetin and RdRp-Remdesivir trajectories. A) displays the first and third eigenvectors, B) shows the second and third eigenvectors, and C) depicts the PC1-PC2-PC3. In the plots, small dots transitioning from white to black represent frames from the RdRp-Remdesivir simulation, while dots changing from white to red represent frames from the RdRp-Quercetin simulation.

Fig 16 displays display the projections of individual trajectories onto the first three eigenvectors. In Fig 16A (PC1 vs PC3), the RdRp-Remdesivir trajectory showed more clustered sampling compared to RdRp-Quercetin, which shows a broader distribution towards throughout simulation run time. In contrast, (PC2 vs PC3) shows minimal overlap between the two trajectories, particularly during first frames of simulation, reflecting a degree of divergence in dynamic behaviour Fig 16B. Finally, 3D projection of PC1, PC2, and PC3 shown in Fig 16C highlights overall structural variability in conformational space between the two systems. In conclusion, the PCA results indicate that the RdRp-Remdesivir system has more constrained and localized motions, while the RdRp-Quercetin system explores a wider range of conformational states, particularly towards the end of the simulation.

### 2.2. *In vitro* assay

#### 2.2.1. *In vitro* RdRp’s inhibition assay

**Fig 17:**
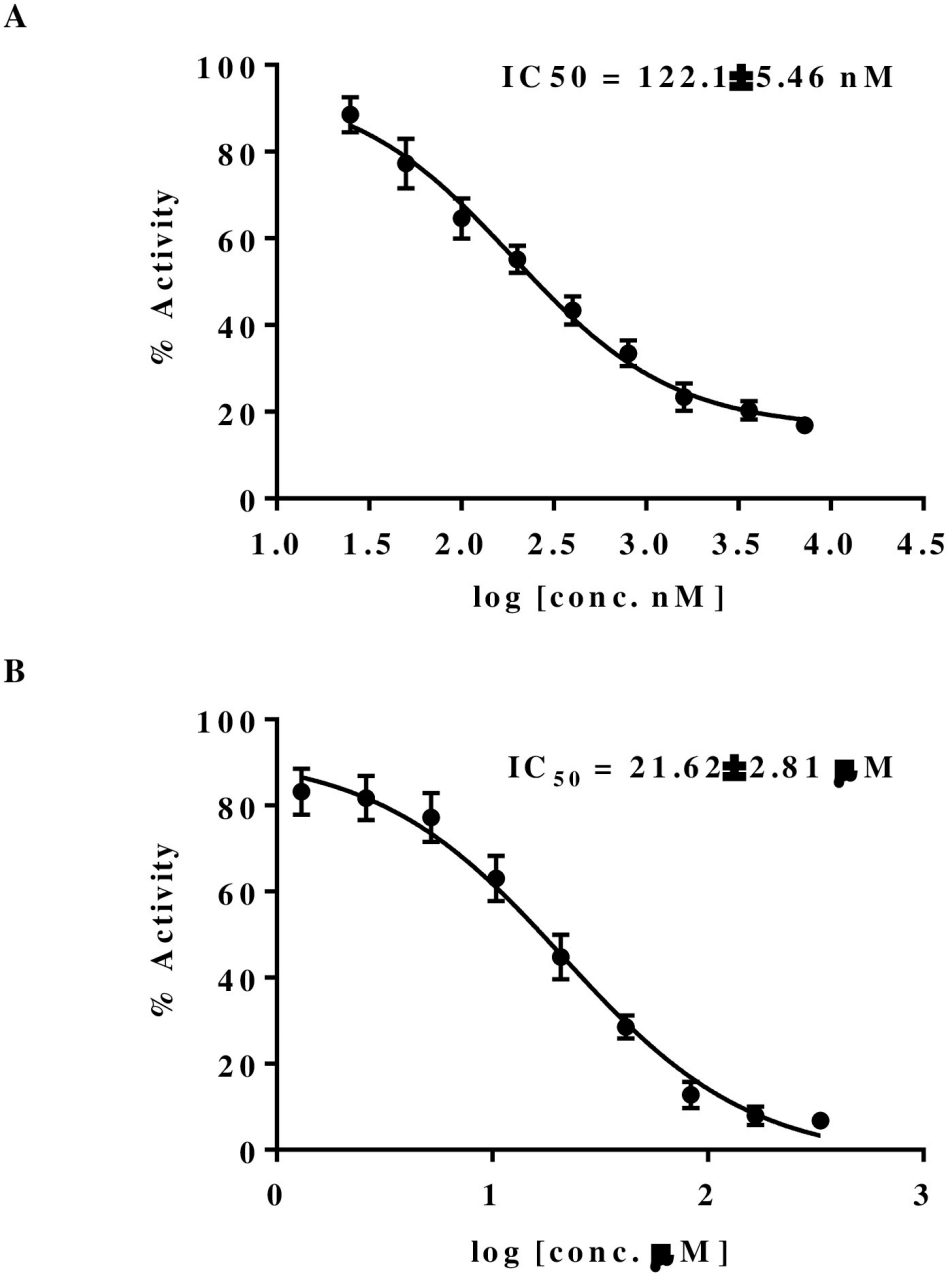
Dose-response curves of RdRp activity inhibition by Quercetin (A) Remdesivir (B).

The *In silico* studies has given us valuable insights into the RdRp-Quercetin complex. These analyses have enhanced our understanding of its binding affinity, dynamic behavior, and energetic interactions compared to the RdRp-Remdesivir complex. However, to confirm these findings, moving to *In vitro* experiments is essential. *In vitro* studies allow for experimental validation of computational predictions, evaluation of biological responses, and verification of binding modes, dynamic behaviors, and energetic interactions. To measure RdRp inhibition, we tested Quercetin and Remdesivir for their IC_50_ values using the RdRp fluorescence kit (SARS-CoV-2 RdRp TR-FRET Assay kit). This assay determines the concentration needed to inhibit 50% of RdRp activity, providing important information on each compound’s potency in blocking the RNA synthesis essential for viral replication. Notably, Fig 17 illustrates that Quercetin displayed an IC_50_ of 248 nM, while Remdesivir exhibited a much higher IC_50_ of 20 μM, indicating a stronger binding affinity and significantly higher potency of Quercetin over Remdesivir. This outcome underscores Quercetin’s promising effectiveness as a strong inhibitor of RdRp. The demonstrated potency in the nanomolar range aligns with clinically relevant levels, highlighting its potential therapeutic value in fighting SARS-CoV-2. This suggests Quercetin could serve as a beneficial alternative or complementary treatment option.

#### 2.2.2. *In vitro* SARS-CoV-2 inhibition assay

**Fig 18:**
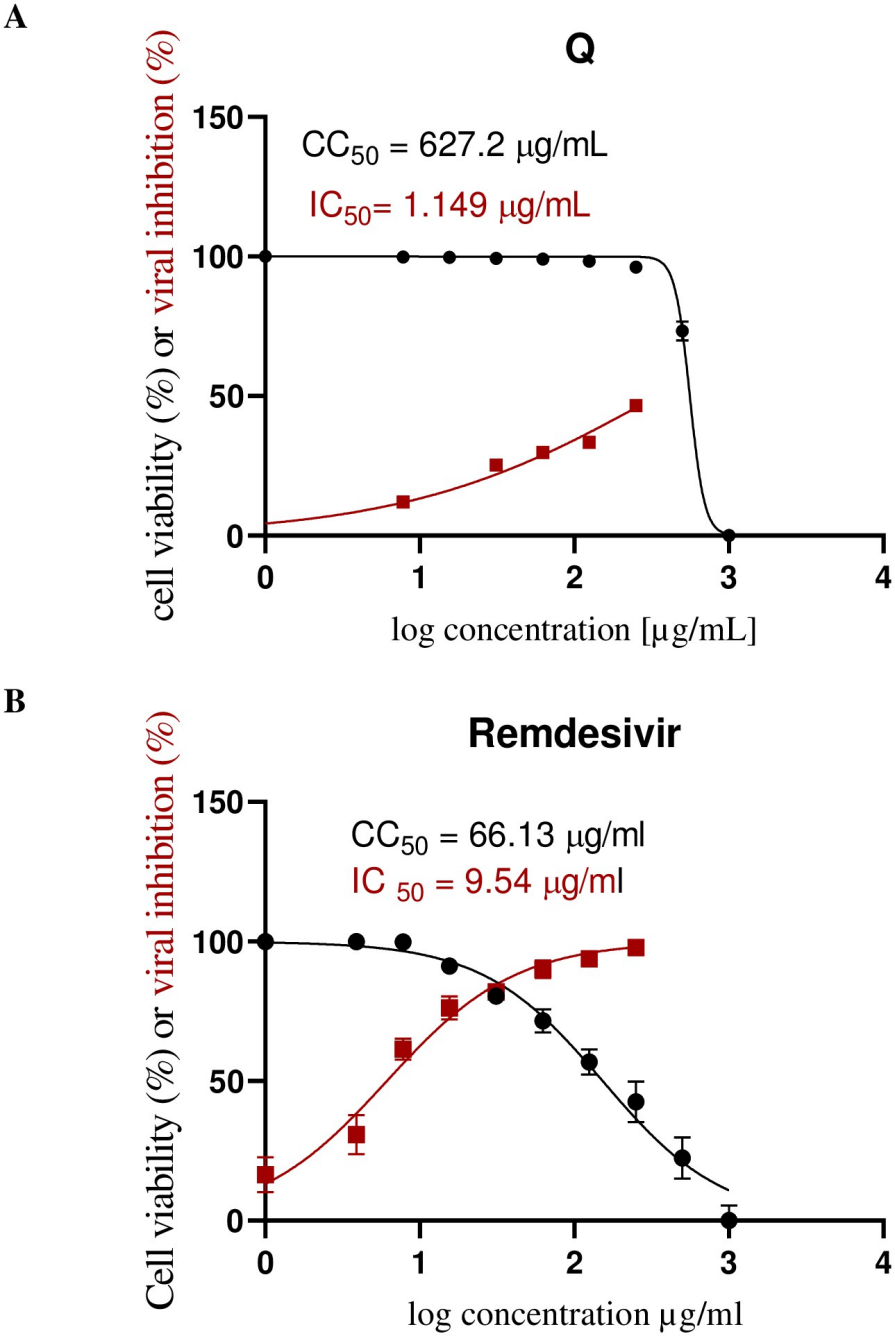
The dose-response curves of Quercetin (A) and Remdesivir (B) against SARS-CoV-2 and their CC_50_ (μg/mL) values. All data are presented as mean ± SD, with n = 3 replicates.

To validate the promising inhibitory effects of Quercetin against RdRp, we assessed its anti-SARS-CoV-2 properties *In vitro* and compared them with Remdesivir. Initially, the cytotoxicity of Quercetin and Remdesivir in Vero E6 cells was evaluated, which serve as host cells for SARS-CoV-2. The MTT assay results indicated that Quercetin has a highly favorable safety profile in a dose-dependent manner, with a CC_50_ value of 376.4 μg/mL (Fig 18A). In contrast, Remdesivir showed a significantly lower CC_50_ of 63.8 μg/mL (Fig 18B), suggesting that Quercetin is safer for these cells compared to Remdesivir. Next, we determined the IC50 values, representing the concentration needed to inhibit 50% of viral replication, for both Quercetin and Remdesivir against SARS-CoV-2 (Fig 18). Remarkably, Quercetin demonstrated an exceptionally low IC_50_ value of 0.476 μg/mL, highlighting its potent inhibitory effect on the virus. In contrast, Remdesivir exhibited a higher IC_50_ value of 10.86 μg/mL, indicating that a comparatively higher concentration is required to achieve the same level of inhibition. These findings underscore Quercetin’s robust anti-SARS-CoV-2 activity and highlight its potential as a promising therapeutic agent against the virus.

Evaluating the selectivity index (SI) values offers important insights into the safety of the compounds. The SI is calculated by dividing the CC_50_ (the concentration causing 50% cytotoxicity) by the IC_50_ (the concentration needed to inhibit viral replication). This ratio shows the safety margin between effective antiviral activity and potential toxicity. In this study, Quercetin has an impressive SI of 791, much higher than Remdesivir’s SI of 6. This higher SI for Quercetin indicates a larger safety margin, suggesting it is a potentially safer option compared to Remdesivir. The favorable selectivity index supports the idea of Quercetin as a promising anti-SARS-CoV-2 treatment with a better safety profile, warranting further exploration and development.

*In silico* and *in vitro* research has shown that Quercetin can disrupt multiple stages of the coronavirus entry and replication process [27], specifically targeting PL^pro^, 3CL^pro^, [50] NTPase/helicase [51], the viral spike protein and human ACE2 [52]. Against the RdRp, Quercetin showed promising in silico inhibition among other 22 metabolites through m molecular docking study [50]. We report here a detailed *in silico* study examining the mechanism of action at the molecular level using MD simulations, PCAT, in addition to molecular docking. Furthermore, we investigated the *in vitro* inhibition potential of Quercetin against RdRp and SARS-CoV-2.

## 3. Experimental

### 3.1. Molecular similarity

The goal was to assess the molecular similarity between Quercetin and nine co-crystallized ligands of SARS-CoV-2 using Discovery Studio 4.0, following established protocols [53, 54]. Initially, the CHARMM force field was applied, and the compound underwent specific preparation steps. Quercetin served as the reference compound, while the co-crystallized ligands acted as the test set. Various molecular properties were examined, including rotatable bonds, rings, aromatic rings, hydrogen bond donor and acceptor atoms, partition coefficient (ALog p), molecular weight (M. Wt), and molecular fractional polar surface area (MFPSA). The S1 File contain additional data, methodologies, and crucial details that provide deeper insights into the experiments and findings of this experiment.

### 3.2. Docking studies

Docking studies utilized the crystal structure of SARS-CoV-2 RdRp (PDB ID: 7BV2) obtained from the Protein Data Bank, using MOE2014 [55, 56]. The RdRp underwent necessary preparation steps, and the structures of Remdesivir and Quercetin were drawn using ChemBioDraw and refined within MOE. Docking involved generating 30 poses for each compound, scoring via ASE, and refinement using a force field. Discovery Studio 4.0 was employed to visualize results, offering insights into potential binding modes within the active site of SARS-CoV-2 RdRp. S1 File provide additional data, methodologies, and crucial details enriching the understanding of the experiments and findings.

### 3.3. MD simulation studies

To evaluate the stability of the Remedisver-RdRp and Quercetin-RdRp complexes [57], GROMACS 2021 software was employed to conduct a 200 ns unbiased MD simulations [58]. Input files were generated with the CHARMM-GUI server, and the analysis employed the CHARMM36m force field for amino acids and the CHARMM general force field (CGenFF) for Quercetin [59, 60]. The S1 File contain additional data, methodologies, and crucial details that provide deeper insights into the experiments and findings of this experiment.

### 3.4. MM-GBSA studies

Binding energies of the Remedisver-RdRp and Quercetin-RdRp complexes were determined using the MM-GBSA method with the gmx_MMPBSA programing [61, 62]. A decomposition analysis evaluated the contribution of each amino acid located within a 1-nanometer radius of ligands to the overall binding affinity. The S1 File contain additional data, methodologies, and crucial details that provide deeper insights into the experiments and findings of this experiment.

### 3.5. ProLIF studies

Ligand-amino acid interactions were thoroughly examined using the ProLIF Python program to identify interacting amino acids and evaluate their significance in stability maintenance [48]. The S1 File contain additional data, methodologies, and crucial details that provide deeper insights into the experiments and findings of this experiment.

### 3.6. PLIP studies

Trajectories were clustered using TTclust to obtain representative frames, and PLIP analyzed and quantified interactions within these frames. Results were visualized in a three-dimensional format using PyMol [49, 63]. The S1 File contain additional data, methodologies, and crucial details that provide deeper insights into the experiments and findings of this experiment.

### 3.7. PCAT studies

PCAT analyzed coordinated motions in MD trajectories using the mass-weighted covariance matrix of a specific atom subset [64, 65]. The S1 File contain additional data, methodologies, and crucial details that provide deeper insights into the experiments and findings of this experiment.

### 3.8. Quercetin

Quercetin was obtained from Thermo Fisher Scientific, Geel, Belgium.

### 3.9. *In vitro* RdRp inhibition assay

Anti-COVID-19 properties of Quercetin and Remdesivir were evaluated using the SARS-CoV-2 RdRp TR-FRET Assay kit, obtained from BPS Bioscience, San Diego, CA 92121, United States measuring ATP incorporation into RNA [66]. The S1 File contain additional data, methodologies, and crucial details that provide deeper insights into the experiments and findings of this experiment.

### 3.10. *In vitro* cytotoxicity assay

Cytotoxicity was assessed via the MTT assay on Vero E6 cells treated with Quercetin and Remdesivir [67]. The S1 File contain additional data, methodologies, and crucial details that provide deeper insights into the experiments and findings of this experiment.

### 3.11. *In vitro* anti-SARS-CoV-2 assay

Vero-E6 cells were infected with SARS-CoV-2 and treated with Quercetin and Remdesivir to assess antiviral activity [68]. The S1 File contain additional data, methodologies, and crucial details that provide deeper insights into the experiments and findings of this experiment.

## 4. Conclusion

In summary, our extensive investigation into the potential inhibitory effects of Quercetin against SARS-CoV-2 RdRp has unveiled promising findings. Through a series of computational analyses encompassing structural similarity assessments, molecular docking simulations, and MD simulations, we have elucidated compelling parallels between Quercetin and Remdesivir, a well-known RdRp inhibitor. Notably, our findings indicate that Quercetin exhibits robust binding interactions with RdRp, outperforming Remdesivir in terms of binding affinity. Moreover, our *In vitro* assays have provided compelling evidence of Quercetin’s remarkable inhibitory potency against both RdRp and SARS-CoV-2, underscoring its potential as a highly effective therapeutic agent. Importantly, the superior selectivity and safety profile of Quercetin, as evidenced by its viral selectivity index values, further reinforce its promise as a candidate for the development of safe and potent anti-COVID-19 treatments. In conclusion, our comprehensive analysis positions Quercetin as a promising lead compound worthy of further exploration and development in the ongoing quest to combat the COVID-19 pandemic.

## Supporting information

S1 File The detailed methodology for the molecular similarity, the molecular docking, the MD simulations, the ProLIF, the PLIP, the MM-GBSA, the PCAT, and the *In vitro* studies. (PDF)
