# Supplementary material for: Integrated study of Quercetin as a potent SARS-CoV-2 RdRp inhibitor: Binding interactions, MD simulations, and In vitro assays
Source: PLoS One. 2024 Dec 3;19(12):e0312866. doi: 10.1371/journal.pone.0312866 (PMC11614241; doi:10.1371/journal.pone.0312866)
Supplement: S1 File — (PDF) [file pone.0312866.s001.pdf]

**Supporting file**

# Integrated Study of Quercetin as a Potent SARS-CoV-2 RdRp Inhibitor: Binding Interactions, MD Simulations, and In Vitro Assays

Ahmed M. Metwaly [1*], Esmail M El-Fakharany [2,3], Aisha A. Alsfouk[4], Ibrahim M. Ibrahim [5], Eslam B. Elkaeed [6], Ibrahim. H. Eissa [7]

[1] Pharmacognosy and Medicinal Plants Department, Faculty of Pharmacy (Boys), Al-Azhar University, Cairo, Egypt ametwaly@azhar.edu.eg

[2] Protein Research Department, Genetic Engineering and Biotechnology Research Institute (GEBRI), City of Scientific Research and Technological Applications (SRTA-City), New Borg El-Arab City, Alexandria, 21934, Egypt esmailelfakharany@yahoo.co.uk

[3]Pharmaceutical and Fermentation Industries Development Centre (PFIDC), City of Scientific Research and Technological Applications (SRTA-City), New Borg Al-Arab, Alexandria, Egypt.

[4] Department of Pharmaceutical Sciences, College of Pharmacy, Princess Nourah bint Abdulrahman University, P.O. Box 84428, Riyadh 11671, Saudi Arabia aaalsfouk@pnu.edu.sa

[5] Biophysics Department, Faculty of Science, Cairo University. Cairo 12613, Egypt. ibrahim_mohamed@cu.edu.eg

[6] Department of Pharmaceutical Sciences, College of Pharmacy, AlMaarefa University, Riyadh 13713, Saudi Arabia ekaeed@um.edu.sa

[7] Pharmaceutical Medicinal Chemistry & Drug Design Department, Faculty of Pharmacy (Boys), Al-Azhar University, Cairo11884, Egypt ibrahimeissa@azhar.edu.eg

***Corresponding authors:**

**Ahmed M. Metwaly**

Pharmacognosy and Medicinal Plants Department, Faculty of Pharmacy (Boys), Al-Azhar University, Cairo 11884, Egypt. **Email:** ametwaly@azhar.edu.eg

**Content**


| | |
|---|---|
| **Method** | **Molecular Similarity** |
| | **Molecular Docking** |
| | **MD Simulations** |
| | **ProLIF** |
| | **PLIP** |
| | **MM-GBSA** |
| | **PCAT** |
| | ***In vitro* assays** |
| **References** | |

**Molecular Similarity:**

The study aimed to evaluate the molecular similarity between Quercetin and a set of nine co-crystallized ligands associated with SARS-CoV-2, utilizing computational tools from Discovery Studio 4.0. Initially, the molecular structures underwent CHARMM force field analysis to establish consistent computational parameters. Subsequently, meticulous preparation of Quercetin was conducted using the designated "prepare ligand" protocol to ensure suitability for comparative analysis.

In this comparative analysis, Quercetin served as the reference compound, while the group of co-crystallized ligands acted as the test set. To streamline analysis and interpretation, the protocol was fine-tuned to generate a single comprehensive output. Standard default molecular properties were applied for consistency and reliability across the study. The examined co-crystallized ligands included F86, PRD_002214, GWS, X77, VXG, 1N7, SAM, Y95, and XT7.

Various critical parameters were scQuercetinized in both Quercetin, the reference compound, and the nine co-crystallized ligands, covering a range of structural and physicochemical characteristics. These parameters included rotatable bond enumeration, ring and aromatic ring counts, hydrogen bond donor and acceptor atom counts, as well as partition coefficient (ALog p), molecular weight (M. Wt), and molecular fractional polar surface area (MFPSA). This comprehensive exploration aimed to reveal any structural similarities or dissimilarities between the tested compound and known ligands, providing valuable insights into their potential interactions and pharmacological relevance in combating SARS-CoV-2.

**Molecular Docking:**

The crystal structure of SARS-CoV-2 RNA-dependent RNA polymerase (RdRp), obtained from the Protein Data Bank with the PDB ID 7BV2, formed the basis of this meticulous investigation. To facilitate docking studies, the RdRp crystal structure underwent meticulous preparation steps using MOE2014 software. Initially, water molecules were removed, followed by protonation of the selected chain and subsequent energy minimization to optimize conformation. Subsequently, the active site of RdRp was precisely defined for targeted analysis. The molecular structures of Remdesivir and Quercetin were meticulously crafted using ChemBioDraw Ultra 14.0 and saved in MDL-SD format. These files were then imported into MOE, where the 3D structures were further refined through protonation and energy minimization processes. To validate the docking methodology, the co-crystallized ligand was initially docked against the isolated pocket of the active site. The Root Mean Square Deviation

(RMSD) value derived from this process served as a crucial metric, affirming the reliability and accuracy of the subsequent docking experiments. The actual docking of Remdesivir and Quercetin was conducted using the docking option integrated into the compute window, generating 30 docked poses for each compound. Adaptive Simulated Annealing (ASE) was employed for the scoring function, and a force field was applied for refinement. The results from the docking process were meticulously visualized using Discovery Studio 4.0 software, offering insights into the potential interactions and binding modes of Remdesivir and Quercetin within the active site of SARS-CoV-2 RdRp.

**Molecular Dynamics Simulations**

To evaluate the comparative stability of the RdRp and Qurecetine complex with a reference compound (Remdesivir), an unbiased molecular dynamics (MD) simulation was performed using the GROMACS 2021 software, spanning a duration of 200 nanoseconds (1). The CHARMM-GUI server's solution builder module was employed to generate the necessary input files (2–5). For each complex, the system was immersed in a solvent environment contained within a cubic box measuring 12.5 nanometers in each direction. The solvation process incorporated the use of the transferable intermolecular potential 3 points (TIP3P) water model, along with the inclusion of a padding region that extended 1 nm from the furthest atom in the system. To achieve system neutrality, NaCl ions were introduced at a concentration of 0.154 M. The CHARMM36m force field was employed to determine the amino acid parameters for the RdRp protein, as well as for the TIP3P water model and the neutralizing ions. The CHARMM general force field (CGenFF) was utilized for parameterizing the Qurecetine and Remdesivir molecules.

During the simulation, periodic boundary conditions (PBC) were applied in all three dimensions. To prevent atomic collisions, the potential energy was minimized. The minimization process was considered converged when the maximum applied force on any atom reached a value below 100 kJ/(mol.nm) or after 100,000 minimization steps were completed. Two stages of equilibration were performed to establish thermal and pressure equilibrium within the systems. In the initial equilibration phase, the NVT ensemble was employed, utilizing the Velocity Rescale method to attain an average temperature of 310 K. Subsequently, the NPT ensemble was employed in the following phase, along with the Berendsen barostat and velocity rescaling, to maintain an atmospheric pressure of 1 atm and an average temperature of 310 K (6). During the 200 ns production run, the NPT ensemble was utilized,

with temperature and pressure control achieved via the Nose-Hoover thermostat and Parrinello-Rahman barostat, respectively. The temperature was maintained at 310 Kelvin, while the pressure was kept at 1 atmosphere (7). The LINear Constraint Solver (LINCS) algorithm was implemented to impose constraints on the lengths of hydrogen-bonded atoms (8). Electrostatic calculations were performed using the Particle Mesh Ewald (PME) method with a threshold of 1.2 nm (9). The leap-frog integration scheme was employed to numerically integrate the Newtonian equations of motion, using a time step of 1 femtosecond during equilibration and 2 femtoseconds for the production run. Throughout the simulation, a total of 2,000 frames were captured at intervals of 0.1 ns.

After recentering the protein within the periodic box to restore its integrity using the gmx trjconv tool, an extensive analysis of the trajectory was carried out using VMD TK scripts (10). Various calculations were performed to evaluate the Root Mean Square Deviation (RMSD) for both RdRp and each compound. Additionally, several other structural features were examined, including Root Mean Square Fluctuation (RMSF), Radius of Gyration (RoG), Solvent Accessible Surface Area (SASA), changes in the number of hydrogen bonds formed between the ligand and RdRp, and the distance between the ligand and the protein's center of mass. Each frame of the trajectory was meticulously scrutinized to investigate and characterize ligand-amino acid interactions.

**ProLIF and PLIP Studies:**

This analysis was facilitated by employing the Protein-Ligand Interaction Fingerprints (ProLIF) Python program, which allowed for the identification of interacting amino acids and the assessment of their relative significance in maintaining stability (11). Subsequently, each trajectory was subjected to clustering using TTclust to obtain representative frames for each cluster. The Protein-Ligand Interaction Profiler (PLIP), commonly referred to as PLIP, was utilized for the analysis and quantification of the interactions observed within these frames. The resulting information was then presented in a three-dimensional format using a .pse file, which could be visualized using PyMol (12,13).

**Binding free energy calculation using MM-GBSA:**

The determination of the ligand's binding energy was carried out using the MM-GBSA methodology in the gmx_MMPBSA program. Additionally, a decomposition analysis was performed to assess the contribution of each amino acid located within a 1-nanometer radius of the ligand to the overall binding (14,15). The selected parameters included an ionic strength

of 0.154 M and a solvation technique (igb) value of 5. The internal dielectric constant was set to 1.0, while the external dielectric constant was set to 78.5. Mathematically, the MM-GBSA method can be described by equation 1.

$$\Delta G = < \text{Gcomplex} - (\text{Greceptor} + \text{Gligand}) > \qquad \text{Equation 1}$$

Where < > represents the average of the enclosed free energies of complex, receptor, and ligand over the frames used in the calculation. In our approach, we used the whole trajectory (a total of 2000 frames). Different energy terms can be calculated according to Equations 2 to 6 as follows:

$$\Delta \text{Gbinding} = \Delta H - T\Delta S \qquad \text{Equation 2}$$

$$\Delta H = \Delta \text{Egas} + \Delta \text{Esol} \qquad \text{Equation 3}$$

$$\Delta \text{Egas} = \Delta \text{Eele} + \Delta \text{EvdW} \qquad \text{Equation 4}$$

$$\Delta \text{Esolv} = \text{EGB} + \text{ESA} \qquad \text{Equation 5}$$

$$\text{ESA} = \gamma.\text{SASA} \qquad \text{Equation 6}$$

Where:

$\Delta H$ is the enthalpy which can be calculated from gas-phase energy ($E_{gas}$) and solvation-free energy ($E_{sol}$). $-T\Delta S$ is the entropy contribution to the free binding energy. $E_{gas}$ is composed of electrostatic and van der Waals terms; $E_{ele}$, $E_{vdW}$, respectively. $E_{sol}$ can be calculated from the polar solvation energy ($E_{GB}$) and nonpolar solvation energy ($E_{SA}$) which is estimated from the solvent-accessible surface area (16,17).

**Principal Component Analysis:**

Principal Component Analysis (PCA) was implemented to examine the coordinated movements within molecular dynamics (MD) trajectories by analyzing the mass-weighted covariance matrix (C) of a specific subset of atoms. In this investigation, PCA was utilized to evaluate the mobility of alpha carbons in amino acid chains A:Asp40 to chain C:Leu55, excluding the terminal amino acids (18). To ensure alignment consistency, the reference frame for individual trajectories was selected as the final frame of the equilibrium stage within each trajectory. In the case of combined trajectories, the reference structure was chosen as the final frame of the RdRp-Remdesivir complex system after it reached equilibrium. By performing diagonalization on the covariance matrix C, PCA identifies the most appropriate eigenvectors

to capture atomic motions. The eigenvalues associated with each eigenvector indicate that the first principal component possesses the highest value, while subsequent principal components exhibit diminishing values, indicating reduced motion. To accomplish this, the GROMACS software was employed, utilizing the gmx covar program to diagonalize the C matrix, followed by analysis using the gmx anaeig program.

The determination of the optimal size of the essential subspace involved several metrics. First, the cumulative sum of eigenvalues in ascending order of eigenvectors was calculated. Second, the scree plot was analyzed to identify the point at which the eigenvalues versus eigenvector indices exhibited the steepest slope reduction. Lastly, the distribution of eigenvectors was considered, recognizing that non-random eigenvectors deviate from a Gaussian distribution.

We computed the cosine content (ci) of each eigenvector of the C matrix, which may take values ranging from 0 (no cosine) to 1 (perfect cosine). The following is the cosine content equation:

$$c_i = \frac{2}{T} \left(\int \cos\,(i\pi t\; p_i(t)\; dt\right)^2 \left(\int p_i^2\,(t)\; dt\right)^{-1}$$

Where T is the time of the simulation. Abnormally large ci values, which represent random motion, are related to insufficient sampling. When the cosine content of the first few PCs is near 1, the behavior of proteins on a large scale is analogous to diffusion. Accordingly, the first 10 PCs were used to calculate their cosine content (19–21).

After aligning the combined trajectories of the reference complex and the RdRp-Qurecetine complex with the initial configuration obtained during the equilibration of the reference complex, a new covariance matrix was constructed for the combined trajectories. Consequently, each trajectory was projected onto this new C matrix, facilitating a straightforward comparison of frames within the reduced essential subspace. Utilizing various combinations of eigenvector pairs, we projected each trajectory onto the first three eigenvectors that comprised the essential subspace. This allowed us to evaluate the degree of similarity and sampling between the two trajectories.

**In vitro RdRp inhibition Assay**

The potential anti-COVID-19 properties of Quercetin were evaluated by examining its ability to inhibit the activity of the SARS-CoV-2 RNA-dependent RNA polymerase (RdRp). The inhibition of SARS-CoV-2 RNA synthesis was assessed using the commercial

fluorescence kit designed for RdRp activity (SARS-CoV-2 RdRp TR-FRET Assay kit). This kit was employed to investigate the inhibitory effects of Quercetin on RdRp, a crucial enzyme involving NSP7, NSP8, and NSP12 proteins in the life cycle of SARS-CoV-2. The kit components include Digoxigenin-labeled RNA duplex, biotinylated ATP, RdRp assay buffer (comprising two components plus DTT), and purified RdRp mixture proteins. The assay measures the amount of biotinylated ATP incorporated directly into the double-stranded RNA substrate, with the increase in the TR-FRET signal inversely related to ATP incorporation into RNA. The kit enables the detection of RdRp activity in just two steps: the enzyme in the reaction mixture is first treated with the test substance, and after the addition of dye- and eu-labeled acceptor and antibody, the TR-FRET signal is then read. This method provides a straightforward way to assess the impact of Quercetin on RdRp activity.

**In vitro cytotoxicity using MTT assay**

The cytotoxic effect of the prepared compounds against normal Vero E6 (SARS-CoV-2 host cells) was determined using the (3-(4,5-dimethylthiazol2-yl)-2,5-diphenyltetrazolium bromide) tetrazolium reduction (MTT) assay. Vero E6 cells ($1.0 \times 10^4$ cells/well) were cultured into 96- well tissue culture plates in supplemented DMEM medium (SERANA, Germany) with 10% fetal bovine serum (FBS, Gibco, USA) and 1 % Pen/Srep mixture (Lonza, USA). After incubation for overnight, Vero E6 cells (accession number: KC869678.4) were treated with different doses ranged from 3.75 to 1000 µg/mL of each compound (P, Q and R) and cultured in 5% $CO_2$ incubator in triplicates. 72 h later, the supernatant was discarded, and cell monolayers were washed with sterile 1x phosphate buffer saline (PBS) 3 times and MTT solution (20 µl of 5 mg/mL stock solution) was added to each well containing 180 µl BPS, pH 7.2 and incubated at 37 °C for 4 h. After medium aspiration, the formed formazan crystals in each well were dissolved with 200 µl of acidified isopropanol (0.04 M HCl in absolute isopropanol = 0.073 ml HCL in 50 ml isopropanol). Absorbance of formazan solutions was measured at λmax 570 nm with 620 nm as a reference wavelength using a multi-well plate reader. The percentage of cytotoxicity compared to the untreated cells was determined with the following equation: The plot of % cytotoxicity versus sample concentration was used to calculate the concentration which exhibited 50% cytotoxicity ($CC_{50}$).
% cytotoxicity = ((absorbance of cells without treatment-absorbance of cells with treatment) X 100)/ (absorbance of cells without treatment).

**In vitro antiviral activity against SARS-CoV-2**

In 96-well tissue culture plates, $2.4\times10^4$ Vero-E6 cells were distributed in each well and incubated overnight at a humidified 37°C incubator under 5% $CO_2$ condition. The cell monolayers were then washed once with 1x PBS and subjected to virus adsorption (hCoV-19/Egypt/NRC-03/2020 (Accession Number on GSAID: EPI_ISL_430820)) for 1 h at room temperature (RT). The cell monolayers were further overlaid with 50 µl of DMEM containing varying concentrations of the test sample (0.0-500 µg/mL, 2-fold)). Following incubation at 37°C in 5% CO2 incubator for 72 h, the cells were fixed with 100 µl of 4% paraformaldehyde for 20 min and stained with 0.1% crystal violet in distilled water for 15 min at RT. The crystal violet dye was then dissolved using 100 µl absolute methanol per well and the optical density of the color is measured at 570 nm using Anthos Zenyth 200rt plate reader (Anthos Labtec Instruments, Heerhugowaard, Netherlands). The $IC_{50}$ value of each compound is that required to reduce the virus-induced cytopathic effect (CPE) by 50%, relative to the virus control and calculated from the non-linear regression curve-fit analysis using Graph Pad Prism 7.0. Untreated viral infected Vero-E6 cells were included as a negative control, whereas remdesivir as a standard drug control was used as a positive control for antiviral assays.

References:


1. Abraham MJ, Murtola T, Schulz R, Páll S, Smith JC, Hess B, et al. GROMACS: High performance molecular simulations through multi-level parallelism from laptops to supercomputers. SoftwareX. 2015;1:19–25.

2. Brooks BR, Brooks III CL, Mackerell Jr AD, Nilsson L, Petrella RJ, Roux B, et al. CHARMM: the biomolecular simulation program. J Comput Chem. 2009;30(10):1545–614.

3. Jo S, Cheng X, Islam SM, Huang L, Rui H, Zhu A, et al. Chapter Eight - CHARMM-GUI PDB Manipulator for Advanced Modeling and Simulations of Proteins Containing Nonstandard Residues. In: Karabencheva-Christova T, editor. Biomolecular Modelling and Simulations. Academic Press; 2014. p. 235–65. (Advances in Protein Chemistry and Structural Biology; vol. 96).

4. Jo S, Kim T, Iyer VG, Im W. CHARMM-GUI: A web-based graphical user interface for CHARMM. J Comput Chem. 2008;29(11):1859–65.

5. Lee J, Cheng X, Swails JM, Yeom MS, Eastman PK, Lemkul JA, et al. CHARMM-GUI input generator for NAMD, GROMACS, AMBER, OpenMM, and CHARMM/OpenMM simulations using the CHARMM36 additive force field. J Chem Theory Comput. 2016;12(1):405–13.

6. Bussi G, Donadio D, Parrinello M. Canonical sampling through velocity rescaling. J Chem Phys. 2007;126(1):14101.

7. Evans DJ, Holian BL. The nose–hoover thermostat. J Chem Phys. 1985;83(8):4069–74.

8. Hess B, Bekker H, Berendsen HJC, Fraaije JGEM. LINCS: a linear constraint solver for molecular simulations. J Comput Chem. 1997;18(12):1463–72.

9. Essmann U, Perera L, Berkowitz ML, Darden T, Lee H, Pedersen LG. A smooth particle mesh Ewald method. J Chem Phys. 1995;103(19):8577–93.

10. Humphrey W, Dalke A, Schulten K. VMD: visual molecular dynamics. J Mol Graph. 1996;14(1):33–8.

11. Bouysset C, Fiorucci S. ProLIF: a library to encode molecular interactions as fingerprints. J Cheminform. 2021;13(1):1–9.

12. Salentin S, Schreiber S, Haupt VJ, Adasme MF, Schroeder M. PLIP: fully automated protein–ligand interaction profiler. Nucleic Acids Res. 2015;43(W1):W443–W447.

13. Tubiana T, Carvaillo JC, Boulard Y, Bressanelli S. TTClust: a versatile molecular simulation trajectory clustering program with graphical summaries. J Chem Inf Model. 2018;58(11):2178–82.

14. Valdés-Tresanco MS, Valdés-Tresanco ME, Valiente PA, Moreno E. gmx_MMPBSA: A New Tool to Perform End-State Free Energy Calculations with GROMACS. J Chem Theory Comput. 2021;17(10):6281–91.

15. Miller III BR, McGee Jr TD, Swails JM, Homeyer N, Gohlke H, Roitberg AE. MMPBSA. py: an efficient program for end-state free energy calculations. J Chem Theory Comput. 2012;8(9):3314–21.

16. Xue W, Yang F, Wang P, Zheng G, Chen Y, Yao X, et al. What contributes to serotonin--norepinephrine reuptake inhibitors' dual-targeting mechanism? The key role of transmembrane domain 6 in human serotonin and norepinephrine transporters revealed by molecular dynamics simulation. ACS Chem Neurosci. 2018;9(5):1128–40.

17. Tuccinardi T. What is the current value of MM/PBSA and MM/GBSA methods in drug discovery? Vol. 16, Expert opinion on drug discovery. Taylor \& Francis; 2021. p. 1233–7.

18. Amadei A, Linssen ABM, Berendsen HJC. Essential dynamics of proteins. Proteins: Structure, Function, and Bioinformatics. 1993;17(4):412–25.

19. Papaleo E, Mereghetti P, Fantucci P, Grandori R, de Gioia L. Free-energy landscape, principal component analysis, and structural clustering to identify representative conformations from molecular dynamics simulations: the myoglobin case. J Mol Graph Model. 2009;27(8):889–99.

20. Maisuradze GG, Leitner DM. Free energy landscape of a biomolecule in dihedral principal component space: Sampling convergence and correspondence between structures and minima. Proteins: Structure, Function, and Bioinformatics. 2007;67(3):569–78.

21. Hess B. Similarities between principal components of protein dynamics and random diffusion. Phys Rev E. 2000;62(6):8438.
